# Emerging Technologies and Vulnerabilities in Older Adults Without Cognitive Impairments: Systematic Review of Qualitative Evidence

**DOI:** 10.2196/69676

**Published:** 2026-02-19

**Authors:** Annachiara Fasoli, Maria De Luca, Giorgia Beretta, Chris Gastmans, Virginia Sanchini

**Affiliations:** 1 Department of Oncology and Hemato-Oncology, University of Milan, Via Santa Sofia 9/1, Milan, 20122, Italy, 39 02 5031 3223; 2 Centre for Biomedical Ethics and Law, Department of Public Health and Primary Care, KU Leuven, Leuven, Belgium

**Keywords:** aged care, emerging technologies, ethical issues, older adults, vulnerability, impairments, PRISMA

## Abstract

**Background:**

Aged care has recently undergone major transformations due to demographic aging and the concomitant need to manage health care costs. New emerging technologies (ETs) have started to play central roles in the daily management of older adults. For these transformations to effectively promote successful and active aging, it is essential to understand the opinions of older adults on the impact that technology can have on their vulnerabilities and aging process.

**Objective:**

This work aims to study the ethically related impact of ETs on cognitively healthy older adults’ vulnerabilities.

**Methods:**

Using the PRISMA (Preferred Reporting Items for Systematic Reviews and Meta-Analyses) guidelines, we conducted a systematic review of empirical (qualitative) evidence exploring the relationship between ETs and older adults’ vulnerabilities as perceived by older adults (older than 65 years) without cognitive impairments. Five major databases (PubMed, Web of Science, Embase, CINAHL, and Philosopher’s Index) were queried on March 1, 2022. After eliminating duplicates, titles, abstracts, and full texts were screened for relevance. Data analysis and synthesis followed the preparatory steps of the coding process detailed in the Qualitative Analysis Guide of Leuven methodology, which involved carefully reading the publications included, identifying significant themes, and constructing conceptual schemes for each paper. The quality of the publications was evaluated by using the Critical Appraisal Skills Program.

**Results:**

A total of 11,631 results were obtained. Eventually, 70 articles were included, and of these, 46 articles had a high level of methodological quality. The remaining 24 articles had moderate quality. ETs appeared to have an ambivalent effect, mitigating some already existing vulnerabilities, and at the same time, worsening already existing vulnerabilities or creating new vulnerabilities. For example, unconventional monitoring techniques (eg, wearables) often mitigated relational vulnerability, helping to maintain independence and remain at home and in one’s community. Conversely, these same devices may negatively affect moral vulnerability, threatening older adults’ privacy linked to data confidentiality.

**Conclusions:**

This systematic review, which focused on the perceptions of older adults without cognitive impairments, enriches the vast literature about the everyday management and care of seniors by exploring the ethical implications of ETs. This research is complementary to another systematic review of qualitative evidence, which analyzed the views of older people with cognitive disorders on the same topic. Although a certain ambivalence in the use of ETs was identified by both population groups, it is interesting how cognitively healthy older adults give more importance to some dimensions of vulnerability, such as the moral and relational ones, which, in the case of cognitively impaired older adults, are not as significant. Two important aspects identified were the respect of privacy and data security, and the perceived risk of control and surveillance linked to the use of monitoring technologies.

## Introduction

### Background

On December 14, 2020, the United Nations General Assembly declared 2021‐2023 the decade of healthy aging [1]. Healthy aging, as described by the World Health Organization (WHO), aims to improve older adults’ lives globally [2,3] by focusing on not only individuals’ functional capacities and residual abilities but also the broader environmental context.

The world population is aging rapidly, with 9% of the global population being older than 64 years [4], and in Europe, 19% of people are older than 65 years [4]. By 2050, these figures will rise to 16% and 28%, respectively. This demographic shift reflects major improvements in public and individual health [5]. However, aging is often associated with increased vulnerability, frailty, psychophysical decline, reduced functional capacity and autonomy, and the onset of chronic conditions or major disabilities. These challenges require health care systems, communities, and individuals to adopt innovative tools and care models to ensure sustainability and long-term manageability [5].

In this context, technology has become a key resource, being increasingly pervasive in society and having a particular significance for aged care. Various terms, for example, AgeTech, ElderTech, SilverTech, and GeronTech [5,6], refer to the health-related industrial sector developing devices that support older adults and formal or informal caregivers. Many emerging technologies (ETs) have entered the market, defined as “radically novel and relatively fast-growing” technologies “characterized by a certain degree of coherence persisting over time and with the potential to exert a considerable impact on the socioeconomic domain(s)” [7]. Some examples considered in this work include wearables (smartwatches, smart bands, and emergency pendants), smart home technologies, assistive robots, and virtual reality [8]. Purely mechanical devices, such as ramps and rails, mobility aids like wheelchairs, and toilet modifications (shower chairs and bath seats), fall outside the scope of this work.

When used in aged care, these technologies are sometimes referred to as “welfare technologies” or “assistive technologies” [9]. Welfare technologies, a subgroup of ETs, can be applied across various social domains (health care, education, work, art, etc) and include technologies intended to enable older adults to remain at home, support independent living, compensate for staff shortages in health care sectors, reduce costs, and enhance self-reliance [9]. Similarly, assistive technologies promote individual functioning and social participation [10]. These technologies support not only healthy aging but also *active aging* [11,12]*,* fostering patient engagement [13], empowerment, and shared decision-making in health care and self-management [14,15]. Such initiatives, by encouraging older adults to actively participate in their own care and preserve their physical, psychological, relational, and emotional capacities, may also reduce health care costs [8] and ease the burden on informal caregivers. Promoting active aging also supports a shift from reactive care to health promotion and disease prevention, aligned with a “salutogenic perspective” [16,17], which emphasizes individuals’ holistic reflection on managing the vulnerabilities and stressors of old age and prioritizes well-being [12]. ETs can thus contribute substantially to *ageing-in-place* initiatives [5,18-22] and, for cognitively healthy older adults, to *successful aging* [21], which is understood as maintaining a life within one’s own community [18].

As aged care evolves, the home may increasingly become the primary setting for care, delaying institutionalization and helping older adults in maintaining routines consistent with their identity. This transition is supported by ETs and do-it-yourself tools, that is, devices that can be managed autonomously by older adults themselves.

The growing integration of ETs in aged care has led to *gerontechnology*, which studies how advances in technology address older people’s needs [23]. Substantial literature has since examined ETs conceptually and philosophically, focusing on their ethical implications in the everyday care of older adults [9,24-28]. Among them, some studies have also explored the perspectives of older adults and carers on the desirable features of ETs [29,30], and numerous quantitative and qualitative studies have analyzed the impact of ETs, especially the ethical challenges they raise [30-34]. Within this body of research, older adult populations are heterogeneous and often categorized based on cognitive functioning. Some studies have focused on individuals with cognitive impairments (eg, mild cognitive impairment, Alzheimer dementia, vascular dementia, Lewy body dementia, and frontotemporal lobar dementia) [35,36], while others have considered cognitively healthy older adults who remain relatively independent [37]. To ensure that transformations in aged care driven by the spread of ETs truly support the well-being and successful aging of all older adults, it is essential to understand how both population groups perceive the impact of ETs on their vulnerabilities and aging more broadly.

### Working Hypothesis

To the best of our knowledge, no study currently provides a systematic overview of the qualitative evidence regarding the perceptions of older adults without cognitive impairments toward ETs in general (across all the different forms, from robots to monitoring technologies), their use, and their ethically related impact, particularly the relationship between ETs and older adults’ vulnerabilities. The novelty of this work lies both in its systematic mapping of all available qualitative evidence in an aging population and its attention to the intersection between ETs and older adults’ vulnerabilities, a narrow and largely undertheorized area within the broader bioethical debate.

Vulnerability in aged care has been analyzed in multiple facets [38]. Six key types of vulnerabilities have been identified: (1) physical vulnerability (PHV), referring to physiological and pathological bodily decline; (2) psychological vulnerability (PV), encompassing emotional, cognitive, and experiential factors affecting mental health; (3) relational or interpersonal vulnerability (RV), rooted in human ontological interdependence and dependence in real-life contexts; (4) moral vulnerability (MV), which positively fosters dignity, respect, and moral preferences, but may also lead to infantilization, depersonalization, and stigmatization if negatively interpreted; (5) sociocultural, political, and economic vulnerability (SPEV), caused by unfair situational factors; and (6) existential or spiritual vulnerability (ESV), relating to intrinsic existential and spiritual conditions that intensify with aging.

However, a gap remains regarding how ETs influence these vulnerabilities. Using these 6 dimensions of vulnerability as a reference and considering their potential interactions with ETs, this study aims to fill this literature gap by analyzing how ETs may affect older adults’ vulnerabilities and by assessing their ethically related impact, understood as contents relating to ethical issues within well-known ethical approaches (eg, principlism and relational care ethics) [39].

## Methods

### Systematic Review Procedure

We carried out a systematic review of qualitative evidence to gain a deeper understanding of the perceptions and experiences of older adults without cognitive impairments with respect to their vulnerabilities in relation to the use of ETs. Systematic reviews of qualitative evidence are methodologically rigorous reviews presenting an up-to-date, comprehensive overview of qualitative studies, that is, semistructured interviews, focus groups, and informal observations [40].

The analysis followed several steps. First, we formulated the research questions. Second, we conducted a systematic search of the literature, consisting of an electronic database search and a “snowballing” process. Third, we identified relevant publications on the basis of well-defined inclusion and exclusion criteria. Lastly, published research results, which met the inclusion criteria, were analyzed and synthesized, creating conceptual schemes in order to identify and define the ethical arguments in response to our research questions.

### Research Questions

We formulated the following interrelated research questions:

1. What is the ethically related impact of ETs on cognitively healthy older adults’ vulnerabilities?
2. What suggestions or strategies do cognitively healthy older adults provide for addressing vulnerabilities related to ETs?

### Literature Search

We developed 3 groups of search terms that guided us in formulating the aforementioned research questions (Multimedia Appendix 1). Group 1 consisted of terms that refer to the type of participant, namely, cognitively healthy older adults. Group 2 consisted of terms that pertain to technology and more specifically to ETs. The terms reported in Groups 1 and 2 were identified on the basis of two previous works [8,41]. We used the expressions “social robots” and “assistive robots” (instead of “social and assistive robots”) to intercept as many results as possible. Group 3 consisted of terms that refer to the concept of vulnerability or similar concepts (ie, frailty, fragility, frailness, acceptance, attitude, concerns, discomfort, distress, ethical issues, and ethics). Indeed, as already stated, when associated with the aging population, the concept of vulnerability has a specific connotation, represented in the 6 dimensions presented earlier in the text. However, since there may be some papers that refer, in the content of their reflections, to 1 of the 6 dimensions of vulnerability while nonetheless using a different framing or different expression to refer to it, other terms (eg, distress and discomfort) were added. The keywords “acceptance,” “attitude,” and “concerns” were used for similar reasons. In qualitative studies involving older adults, ethical issues are often not explicitly presented to participants during interviews, focus groups, or other types of approaches. At the most, these issues may be inferred from the broad questions posed to seniors, who are typically asked simpler questions about their adoption of ETs, their concerns, the perceived benefits and drawbacks, the usability, and similar topics.

The 3 groups of concepts were gathered and expressed according to the properties of each major database (Multimedia Appendix 2). The terms and the search strings were chosen and devised by the first author (AF) in consultation with the other authors (VS and CG). The following 5 electronic databases were queried: PubMed, Web of Science, Embase, CINAHL, and Philosopher’s Index. These databases cover the literature in biomedicine, bioethics, philosophy, and (medical) anthropology.

Database queries were conducted on March 1, 2022, and studies not available in English were excluded. Multimedia Appendix 2 presents the number of results returned using the search terms. We used EndNote (version X9; Clarivate Analytics) reference library software to organize the citations of the identified papers, and duplicates were manually deleted.

### Inclusion and Exclusion Criteria

For publications to be included in our systematic review and appraisal, they had to meet a predetermined set of inclusion and exclusion criteria (Multimedia Appendix 3). Screening of the articles was not limited by publication date.

As for the population under study, we included two categories of contributions in our selection of papers: (1) studies using standardized tests to assess cognitive function (eg, Mini Mental State Examination and Montreal Cognitive Assessment) and (2) studies that, while not using formal cognitive assessments, explicitly stated that participants were capable of understanding the study and providing informed consent. In these cases, participants were considered to be not only legally competent (ie, able to consent) but also cognitively competent, as explicitly stated or implied by the authors of selected contributions.

The first (AF) and last (VS) authors screened the titles, abstracts, and full texts of identified papers according to these criteria. The abstract screening was performed separately by the first (AF) and last (VS) authors to make sure that the selection criteria were applied consistently. For 87.3% (3077/3526) of the abstracts, the authors agreed on the items to be included or excluded. For questionable abstracts (449/3526, 12.7%), the first 2 authors (AF and MDL) discussed the candidate abstracts until an agreement was reached.

If the full text of an article was not available, the first author or corresponding author of that article was contacted via email to request a PDF copy. To ensure the search was exhaustive, the “snowball technique” was applied to the reference lists of eligible publications to identify additional potentially relevant publications. The search process was performed according to the PRISMA (Preferred Reporting Items for Systematic Reviews and Meta-Analyses) statement [42]. The PRISMA checklist is provided in Checklist 1.

All the included publications were from the qualitative research literature; thus, they provided insights on the concept of vulnerability and/or related concepts (ie, frailty, frailness, and fragility) in relation to ETs, as expressed by older adults without cognitive impairments during semistructured interviews or focus groups, or in other settings (eg, informal observations).

The final list of included publications and the descriptions of the characteristics of the included publications are presented in MultimediaAppendices 4  5, respectively.

### Quality Appraisal

The quality of the included publications was evaluated by using the Critical Appraisal Skills Program (see the Methodological Quality section). The second author (MDL) assessed each full-text article and constantly discussed the assessments with the first author (AF), who also carefully read and assessed the full texts. When doubts arose, discussions were held until a consensus was reached. This appraisal tool allowed the identification of studies with a high, medium, or low methodological quality. None of the studies were excluded based on methodological quality, because after careful verification, they were all found to be sufficiently adequate.

### Data Extraction and Synthesis

#### Procedure

For data extraction and synthesis, we decided to follow the 5 preparatory stages of the coding process detailed in the Qualitative Analysis Guide of Leuven (QUAGOL) [43]. In the first stage, the second author (MDL) thoroughly read and reread all the included publications to familiarize herself with the data and to identify the significant themes described. Ethically related content was identified based on themes and concepts present in a previously completed review of argument-based ethics literature [39] and those stemming from the relational care ethics approach as operationalized in the dignity-enhancing care model of bioethics [44]. In the second stage, the first (AF), second (MDL), and last (VS) authors developed a narrative summary of the highlighted parts. In the third stage, for each publication, a conceptual scheme was created. This scheme summarized the various concepts that are important to answer the research questions. An example of a conceptual scheme is illustrated in Multimedia Appendix 6. Each conceptual scheme was examined separately by the authors (AF, MDL, and VS) to verify that it corresponded to each selected publication. The authors discussed conceptual schemes until they agreed on adequate content. In the fourth stage, these individual conceptual schemes were compared to verify that the content of the conceptual schemes reflected the most important concepts to answer the research questions. All conceptual schemes were transformed into a global scheme that showed the ethics-related impact of ETs on the dimensions of aged care vulnerabilities (Multimedia Appendix 7), as gleaned from the perceptions and experiences of older adults. An iterative analysis and a check between this scheme and the previous stages of QUAGOL were conducted to ensure that the scheme was consistent with the included papers. In the last stage, as reported in this review, we prepared a description of the results.

#### Categories of ETs

The ETs used in the qualitative studies under review were organized in this work according to the categories proposed in a previous publication [8]. Conventional monitoring techniques (CMTs) are those technologies that rely on traditional devices, such as oximeters or blood pressure monitors, to continuously observe older adults’ physiological and physical parameters and keep track of their condition through technological platforms [8,45-47]. On the other hand, unconventional monitoring techniques (UNMTs) introduce innovative technology components, such as body or environmental sensors, that allow more pervasive monitoring, and they comprise, for instance, smart home technologies or ambient intelligence [48,49]. Virtual reality techniques (VRTs) refer to headsets that create 3D environments in which older adults are “immersed” [50,51]. Finally, socially assistive robots (SARs) are robots that are able to interact with older adults and provide physical or cognitive assistance [52,53].

#### Conceptual Framework

In conducting data extraction and synthesis, we adopted a 2-fold strategy (top-down and bottom-up) to assess the ethically related impact of ETs on vulnerabilities. The top-down approach, a deductive and theory-driven method, helped guide and focus our research questions. The approach originally set out was relational care ethics, which emphasizes the moral significance of care relationships and human dependencies. This approach was operationalized in a dignity-enhancing model of care [44] where both dignity and vulnerability played central roles. We also relied on a framework of macro-themes and key issues derived from the relevant literature [8,24,26,39]. This interpretative lens, encompassing several sensitive ethical concerns, such as security, privacy, and autonomy, was applied, refined, and adapted to analyze the selected studies and to assess their ethical impact in relation to older adults’ vulnerabilities.

The concept of vulnerability itself was informed by a recent systematic review of argument-based literature [38], which proposed a taxonomy we adopted to present ethically related findings. This allowed for a more analytical understanding of how ETs interact with both existing and potential vulnerabilities in cognitively healthy older adults.

The bottom-up approach relied on an inductive, data-driven method, seeking to infer underlying meaning directly from participants’ words and expressions.

## Results

### General Descriptions of Included Publications

**Figure 1.**
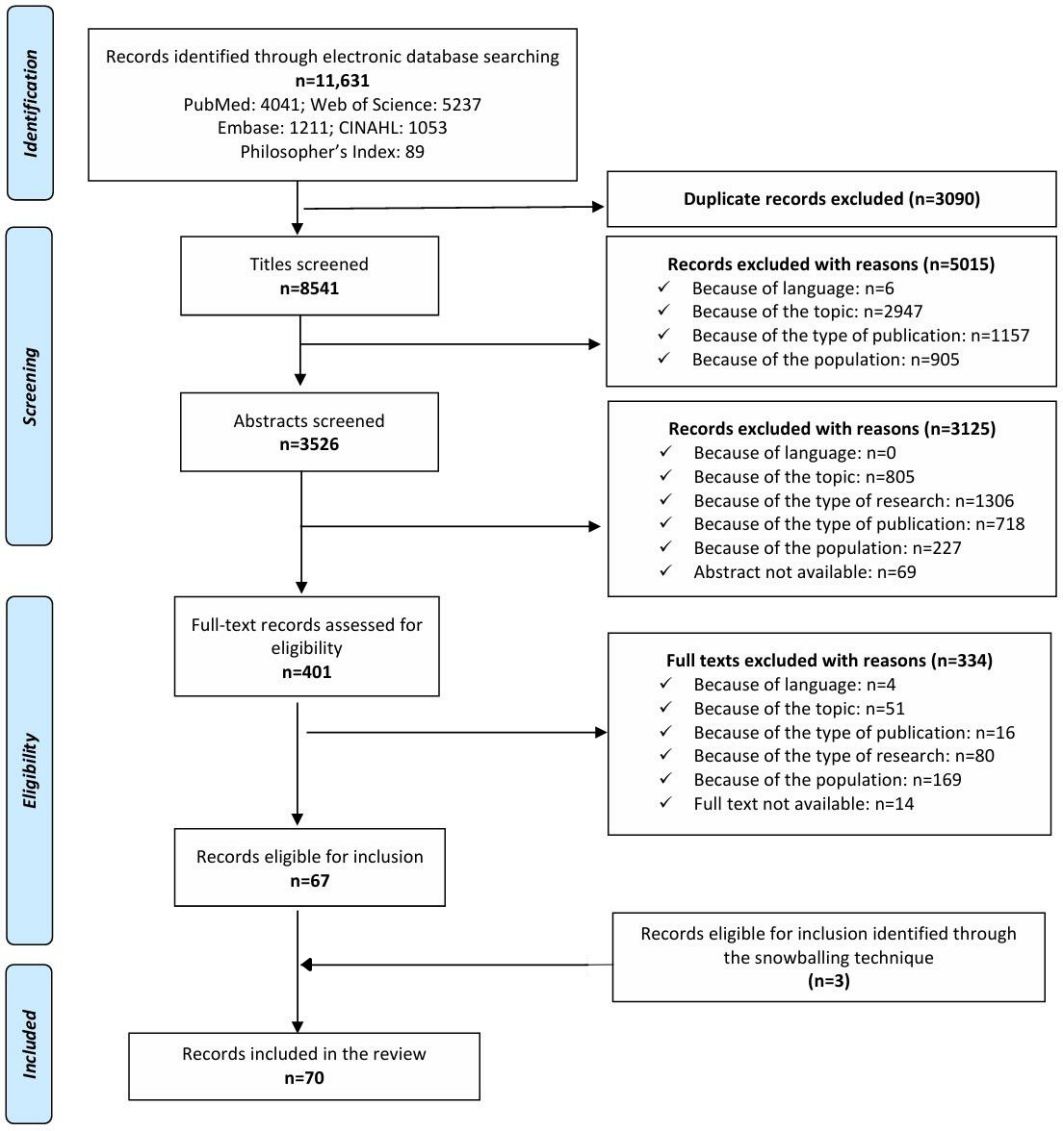
PRISMA (Preferred Reporting Items for Systematic Reviews and Meta-Analyses) flowchart showing the electronic database search, publication identification, and selection process for the included articles.

A total of 70 publications (Multimedia Appendix 4) met our inclusion criteria: 67 were identified from the research queries and 3 were identified through the snowball technique. For a detailed description of the general characteristics of the included papers, see Multimedia Appendix 5. The PRISMA flowchart is provided in Figure 1.

All the included publications were from the qualitative research literature; thus, they provided insights on the concept of vulnerability and/or related concepts (ie, frailty, frailness, and fragility) in relation to ETs (Multimedia Appendix 3), as expressed by older adults without cognitive impairments during semistructured interviews or focus groups and in other qualitative research settings (eg, informal observations) (Multimedia Appendix 5). With respect to the method used to obtain the qualitative data, 61 (87%) studies used semistructured or general interviews (38/70, 54%) or focus groups (23/70, 33%), and 5 (7%) studies used a combination of semistructured interviews and focus groups. Other methods included observations (7/70, 10%), usually in combination with interviews or focus groups, and self-report diaries (1/70, 1%).

Publication years ranged from 2004 to 2022, with 53 (76%) articles published between 2015 and 2022. The Americas, Europe, Australia-Oceania, and Asia are represented in this systematic review, meaning that the included studies had first authors from all these different continents. However, most studies were conducted in Europe (36/70, 51%) and the Americas (22/70, 31%). Within Europe and the Americas, the authors’ affiliations varied widely. For authors based in Europe, the affiliation for 16 (23%) publications was an institution in Britain or Sweden. For authors based in the Americas, the affiliation for 19 (27%) publications was an institution in the United States.

For inclusion in this review, papers had to involve older adults without cognitive impairments in the context of aged care. All the included papers featured older adults aged 65 years or older. More specifically, 20 (28%) studies included older adults aged 70 years or older, and 2 (3%) included older adults aged 80 years or older. Among the included studies, 47 (67%) were unclear about or did not mention the health status of the participants. Moreover, 26 (37%) studies reported the participants’ pathological conditions and/or physical impairments, and 11 (16%) studies indicated cardiovascular pathologies or not-specified chronic conditions.

With regard to technologies, all previously mentioned categories of ETs, except for VRTs, were used in the included publications. The most studied category was that of UNMT, which was reported in 41 (58%) publications. Moreover, 29 (41%) studies focused on the use of environmental sensors (eg, chair and bed sensors, motion sensors, and fall detection sensors) [37,54-81]. In the papers, the authors often referred to these monitoring tools using the term “smart home,” and they less frequently used “ambient intelligence” or “Internet of Things.” In 14 (20%) studies [54-56,67,69-71,78,82-87], the focus was on the use of wearable sensors, such as smartwatches and smart bands, which can collect a variety of information regarding physical activity or vital signs. For example, in 1 study [84], participants used Fitbit, a wearable device that monitors some aspects of the user’s behavior and vital parameters (eg, number of steps, distance covered, calories burned, heart rate, and blood pressure).

CMT was the second most studied category, which was noted in 25 (36%) publications. More traditional and common information and communication technologies, such as mobile phones, smartphones, tablets, and computers, were used to provide health-related services (eg, televisits; teleconsultations; and tips for self-management and self-monitoring of one’s health, well-being, and/or illness) through the use of so-called mHealth apps [88,89]. The use of such ETs in health care is referred to as “telehealth” [80,90,91].

Finally, the least studied category was SAR, which was noted in 16 (23%) publications. These robots, which were described in detail in 9 (13%) studies [30,92-99], can be divided into 3 groups: nonanthropomorphic [92,93,95,98,99], anthropomorphic [30,95], and zoomorphic [95-97]. An example of the first type of SAR is the robotic platform Hobbit [92,99], which is connected to an ambient assisted living environment and provides entertainment, reminders, fall and emergency detection, and prevention services. Regarding the second group, NAO [95] and Alice [30] are humanoid robots, which are mostly used as companion-type robots to interact and communicate with older adults. Among robots with animal features, the most famous one is PARO [95-97], a baby seal robot that can elicit positive emotions. PARO was originally designed for older adults with dementia, but it has also proven to be a valid tool for older adults without cognitive disorders, as noted in 3 (4%) studies included in this review.

The fourth category of ETs, VRT, was not present in any of the included studies, and the possible reason is that it is still a state-of-the-art technology and has a high cost.

### Methodological Quality

The quality assessment results of all included publications are summarized in Multimedia Appendix 8. According to the evaluation carried out, 46 (66%) studies had a high level of methodological quality. On the other hand, the remaining 24 (34%) studies were classified as having moderate quality.

All the papers had a clear statement of the aims of the research, explained the relevance, clarified the methodology followed, and presented the findings explicitly and clearly. In 66 (94%) studies, the researchers also discussed the contribution of the study to existing knowledge and identified new areas of research.

With regard to data collection and recruitment strategies, 61 (87%) studies took appropriate measures, paying adequate attention to ethical issues, such as obtaining ethical approval from ethics committees and informed consent from participants. On the other hand, with regard to data analysis, 22 (31%) studies did not provide an in-depth description of the analysis process.

Finally, the quality appraisal revealed 2 important limitations. First, in 56 (80%) studies, researchers failed to justify the research design and describe how they decided which method to use. Second, in 64 (91%) studies, researchers did not take into consideration the influence they would have on participants and the research project, thereby presenting a potential risk for bias.

### Ethics-Related Analysis

The included articles were analyzed using a 2-fold strategy (top-down and bottom-up; see the Conceptual Framework section).

**Figure 2.**
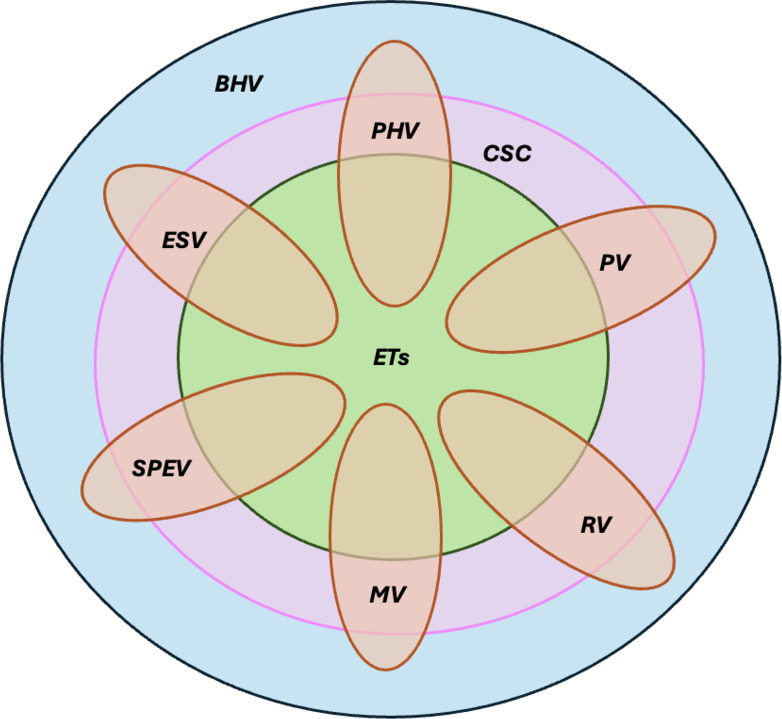
Illustration of the relationship among emerging technologies (ETs), vulnerabilities of older adults, and cultural and social constructs. BHV: basic human vulnerability; CSC: cultural and social constructs; ESV: existential or spiritual vulnerability; MV: moral vulnerability; PHV: physical vulnerability; PV: psychological vulnerability; RV: relational or interpersonal vulnerability; SPEV: sociocultural, political, and economic vulnerability.

Figure 2 provides an illustration to help understand the results of our systematic review in relation to the broader debate surrounding vulnerability in aged care.

Basic human vulnerability refers to the inherent fragility and finitude of human existence, which cannot be eliminated by technological or other means. In response, humanity has developed various cultural and social constructs, including art, health care, social services, education, politics, economics, and technology, to manage both basic and situational vulnerabilities. These constructs are particularly relevant to the 6 dimensions of vulnerability in aging populations. Among them, ETs may impact both basic and situational vulnerabilities; however, some situational vulnerabilities may resist intervention due to their complexity or deep existential roots.

As summarized in Multimedia Appendix 7, the next sections will present the ethically relevant results of the review, analyzing the impact of ETs on the vulnerabilities of older adults, based on what was reported by study participants. Each of the following paragraphs explores 1 of the 6 dimensions of vulnerability, and they are ordered from the most frequently mentioned dimension to the least frequently mentioned one. Finally, the emerging theme of usability, which was not originally present in the taxonomy, has also been presented.

### MV Dimension

MV was the dimension of vulnerability most cited by the qualitative studies in this review, with 43 out of the 70 publications addressing it in its different nuances and meanings.

After analyzing the included papers, we further organized this dimension into the following subcategories: *decisional autonomy and/or self-determination* (noted in 14 studies), privacy linked to *confidentiality* (noted in 24 studies), privacy linked to *control and surveillance* (noted in 19 studies), privacy meant as respect for *integrity and identity* (noted in 9 studies), and *stigmatization and infantilization* (noted in 13 studies).

Older people participating in the studies included in this review emphasized the impacts that ETs have on privacy linked to *confidentiality* and more specifically to the management of personal data collected by devices and their disclosure to third parties (ranging from caregivers to health professionals and to potential strangers). This aspect was consistently framed in negative terms by the interviewees. In fact, in all the 24 studies in which the theme of confidentiality emerged, ETs were believed to have a negative impact, as most participants felt that ETs violated their sensitive and personal data. This was especially true for UNMTs, which were studied in 19 out of 24 publications. In particular, two functions of monitoring technologies seemed to be of great concern for older adults: (1) the recording of images and sounds [54,57,58,60,62,64,70] and (2) the collection of personal data [29,57-59,64-67,71,73,74,77,85,86]. For example, in 1 study [54], older adults felt that the use of a camera was intrusive and violating. One participant said, “I wouldn’t want to feel that somebody was watching my activities.” In another study [57], talking about an IP web camera, participants said that it was “too invasive and would make them feel uncomfortable in their own home.” With regard to a smart speaker, a few participants “expressed concerns it would be used to listen into private conversations.” Considering data collection, storage, and transfer, older adults were concerned about data security and expressed the desire to be protected from possible privacy breaches and from the possible misuse and abuse of their personal data [29,58,59,66,67,71,73,74,77,85,86]. For example, in 1 study [58] that addressed home monitoring techniques, such as wireless motion sensors, in-kitchen temperature sensors, and door contact sensors, a participant stated:

I might be concerned about confidentiality issues, such as a breach of my personal information or potential harms due to my information being exposed to others.…There could be a possibility that a burglar could see my data accidentally and come along after I go out.

In another study [29], in relation to the use of different kinds of technologies based on artificial intelligence, the following statement was made:

If they’re [the technologies] so sensitive, they know three weeks before we know what’s going wrong with our bodies. It seems to me that that kind of information could really be compromised, and seniors could, uh, who are very vulnerable, could really be hoodwinked more easily.

Participants were also concerned about the transfer of data, which they would like to keep confidential and share only with their relatives and/or caregivers [59,63]. In 1 study [59], a participant said:

When you get older, you don’t feel that safe on your feet…and you mean – is it – I have to announce that to everybody?…I think that’s the most private thing that you’re really interfering with.

In another study [63], older adults had concerns even “about family members potentially ‘knowing too much’ and some were concerned that data could be used by families to the detriment of the older adults.”

The second most recurrent topic concerning MV was privacy linked to *control and surveillance*, which was noted in 19 studies. Among these studies, 16 reported that ETs had a mostly negative impact, 2 [77,83] reported that devices had an ambivalent effect on MV, and 1 [100] mentioned that ETs appeared to mitigate MV.

Among the critical issues identified, the most prominent concern associated with ETs in general (CMTs [101] and SARs [30,102]), but especially UNMTs [54,55,59,62,65,70,73,78,79,84]) is surveillance. For example, in 1 study [55], a participant associated this negative feeling of constant monitoring with past traumatizing experiences, when “there were ‘folkpolice’ everywhere watching everyone, and everyone watched everyone else in East Germany… and we don’t want that system here. And, at least not here in our home.” In another study [59], a participant said that “you’d feel like a puppet on a string…I don’t want to be – something watching me.” Finally, in another study [79], participants linked surveillance to the Big Brother Syndrome [103], and the following statement was made:

While they do not mind having the system monitoring them 24/7, the incorporation of a camera into such system would be “too intrusive.” “No, definitely no photos. It’s like big brother, and i’m not having that.”

In 2 studies, UNMTs were reported to have both positive and negative effects. In 1 study [83], which examined a wrist-worn monitoring device, the positive aspect highlighted was the increased sense of safety, resulting from the awareness of being constantly monitored. For example, a participant said:

I feel safe. It must be safer.…In this way they can keep a track of me even when they are not here, which is good because I’m alone here in my house.

This perception of protection, linked to surveillance, was particularly emphasized in relation to falls, as also stated in another study [100]. On the other hand, in these studies, a strong discomfort also emerged, resulting from the feeling of being “watched,” which was perceived as a violation of personal and private space.

Among the included studies, 14 reported that older adults felt that ETs had either a positive or negative impact on their decisional autonomy and/or self-determination. In a positive way, CMTs and UNMTs improved older adults’ decisional autonomy and minimized their loss of control, as they enabled older adults to control certain elements of their house or their life [61,76,104-106]. For example, in some studies [61,76], smart home technologies were really appreciated because these devices provided the possibility to remotely control the environment (eg, lights, television, and doors). With regard to GatorTech Smart Home [61], an older woman liked above all “the ability to command the house to open blinds, doors, lights” and “the ability to see who was at the front door and make the choice to see if wanted to respond rather than having to go over and look through the peephole.”

However, despite this benefit, ETs (CMTs, UNMTs, and SARs) can also interfere in a negative way with older adults’ decisional autonomy, as they direct the life of older adults and make them lose control of their daily activities and decision-making capacity [29,30,55,59,77,94,101,107]. This fear emerges not only in relation to monitoring technologies [29,55,59,77,101,107] but also in relation to the use of SARs [30,94]. Talking about Alice [30], a robot with a small humanoid body and a human-like face, a study mentioned about “negative feelings related to feeling powerless in stopping the perceived technological imperative. Participants felt as if they had no choice but to comply with the evolution of SAR.” One participant said that SARs “cannot take over,” and another claimed that “not everything can just happen because it [the SAR] says it.”

Other frequently mentioned themes in relation to MV were stigmatization and infantilization, which appeared in 13 publications. Twelve publications highlighted how ETs exacerbate feelings of stigmatization and infantilization because older adults associate the use of these devices (mostly UNMTs) with aging and dependency [30,47,55,62,64,74,76,79,80,94,95,104]. For instance, both CMTs and UNMTs [47,62,64,74,76,79,80,104], especially smart home technologies, were despised because the participants believed that these are tools for more vulnerable people with serious disability problems and not devices for healthy individuals like them. Two studies specifically pointed out the phenomenon of depersonalization, as monitoring techniques like sensors [55] and SARs [30] gave older adults the idea of being seen as an *object* rather than a person with individual needs. With respect to the infantilization phenomenon, the issue emerged negatively in relation to the use of SARs. In particular, in 1 study [94], it was stated:

Some participants found the concept of interaction with companion robots demeaning.…Craig was similarly condemnatory of ElliQ: “Crazy. I do think I’m a bit more intelligent than that.”

Likewise, in some studies [30,95], the use of robots was described as childish and dehumanizing.

Only 1 publication showed that ETs could have a positive impact on MV, as, according to the participants of the study, Interaktor, an interactive app for regular reporting of health concerns, allowed health care personnel to take care of older adults as people, reducing depersonalization [46]. Older adults reported a “feeling of being appreciated” and a “feeling of being acknowledged as a valued person.”

Shifting the perspective on privacy again, the last 2 connected themes were integrity and identity. In 9 studies where these themes were mentioned, the negative impact of ETs was emphasized, mostly with reference to UNMTs [54,55,57,59,62,71,77], such as smartwatches, health trackers, chair and bed sensors, motion sensors, and home energy sensors. In 8 publications [54,55,57,59,62,71,94,101], the problem was the perceptions of obtrusion, intrusion, and invasion, which were interpreted as violations of personal space, a kind of “attack,” compromising personal integrity and identity. In addition, in 1 study [60], a participant was very concerned about the potential misuse of her identity, which was caused by the use of “welfare technology” (ie, ambient assisted living).

### RV Dimension

The second most frequently cited dimension of vulnerability was RV, which appeared in 40 out of the 70 publications. These 40 studies focused on interdependence in real-world settings, and none of them reported an impact on the ontological interdependence characterizing human nature, that is, the intrinsic relational and social nature of human beings.

The relationships among older adults, caregivers, personnel, and/or family members appeared in 36 publications. In 19 studies, older adults highlighted only the positive impacts of ETs on RV. In 7 studies, on the other hand, some disadvantages concerning RV emerged. Finally, in 10 studies, participants emphasized the ambivalent effects of ETs on RV.

Most publications showed that ETs can positively influence RV as participants saw ETs as a support to maintain their independence and remain at home and in their own community [57,65,75,81,94,108,109]. This was especially true for UNMTs like smart home technologies. For example, in 1 study [75], with regard to a sensor monitoring system, which included passive infrared motion sensors, magnetic contact sensors on doors and cabinets, and a flush sensor in the toilet, a participant said:

It may be useful for the future.…People can stay at home longer with the help of sensors because there is more supervision.…It is always nice to stay in your own neighborhood, especially for elderly people who have neighbors and friends in their neighborhood.

Another important benefit with regard to RV was the relief from loneliness [61,65,92,94,106,108,110,111]. SARs, in particular, were seen by older adults as pleasant companions to spend time with. In 1 study [94], ElliQ, a nonmobile home assistant robot with moving parts, conversation functions, and a female-sounding voice, was praised by respondents, and it was stated:

…would be comforting in a quiet and lonely household: “It breaks the silence of the day.” For Sarah, the more linguistically sophisticated presence of ElliQ had a positive side, because it was like having a person in the house.

CMTs, such as tablets, smartphones, and laptops, which enable remote communication and video conferencing, were also highly appreciated as ways to connect with members of one’s network and receive practical and emotional support from them [37,89,91,98,106,107,112]. In 1 study [89], a participant’s point of view on the use of mHealth apps was reported:

You’ll [the doctor] assist me in keeping an eye on it [the remedy] and reporting back through the app and then I can connect with a trusted advisor just through the app – that would be great. Convenient.

In another study [112], an interviewee made the following statement about tablets (such as iPad and Kindle Fire): “I feel more informed; I feel I’m in more contact with my family.”

In other publications, however, ETs were reported to have a negative impact on the relationships among older adults, caregivers, personnel, and/or family members. According to 1 study [73], UNMTs like ambient assisted living might reduce the independence of older adults, and a female participant stated:

I think it’s very intrusive and I think people who are over 70 as I am would find it just one step down from you making dependent on somebody…If you feel you’ve got your independence…you know…I think it would be own step down…yes I didn’t like it.

Sometimes ETs were despised because they can replace human interaction and reduce “face-to-face care” [80,90,100,101], increasing the sense of loneliness [101,113]. Interestingly, ETs were considered problematic because they might generate misunderstandings and mistrust among people [113,114]. For example, in 1 study [113], with regard to the use of a tablet-based communication app allowing asynchronous multimedia communication, it was stated:

Six participants verbalized that the technology created family tensions.…Our older people preferred asynchronous communication, to send audio messages, and to receive text messages, but family preferred synchronous communication and video and photo messages. Participants were “disappointed” with relatives that instead of replying to their messages via the app called them on the telephone.

Finally, as anticipated, in 10 studies with regard to RV, older adults expressed their opinions on ETs in ambivalent terms. ETs appeared to enable older adults to maintain and improve their autonomy and independence in daily activities [30,67,72,77,104,105], but the use of these same tools raised their fear of losing residual autonomy and abilities [76,95,104], that is, the set of psychophysical capacities and functions that are not (for the time being) affected by the deterioration that normally characterizes human aging. Moreover, while ETs appeared to improve contact and provide support [30,46,66], which can reduce feelings of loneliness [30,76,95], they may exacerbate this same loneliness [105] and raise fears of a gradual loss of human interaction [30,46,66,67,72,77].

With regard to human-robot interaction, in 4 studies [93,97,108,115], older adults only emphasized the positive aspects of using ETs. In 4 other studies [71,91,92,100], however, only critical issues emerged. In 3 studies [30,94,95], older adults’ opinions were ambivalent. Paradoxically, the use of SARs was appreciated for contradictory reasons. On the one hand, participants appreciated the resemblance of robots to living beings (human beings [95] or animals [94]). For example, in 1 study [94], talking about Biscuit, an animal-like robot, an interviewee said that it showed compassion and “a dog-like affection,” and the interviewee made the following comment:

You could touch Biscuit.…It’s got eyes you can look in. So, you get some sort of empathy back, or feeling back, the way it tilts his head when you talk to him.…More like a real dog.

In 1 study [95], with regard to home robotic devices, it was mentioned that “participants reacted to the nonverbal communication capabilities of some robots, stating: It’s like having another person in the flat that would communicate with me.” However, other older adults held the opposite view, and it was stated that robots are more appreciable because they do not have the “defects” of living beings, both human [30,108] and animal [97]. For instance, in 1 study [108], the following statement was made: “the fact that the robot could not ‘speak, offend, or get angry like a human being’ was mentioned as an advantage of this special companion by one participant.” Likewise, in another study [97], PARO, a baby seal robot, was appreciated because, as a participant stated, “you don’t have to feed him or clean his litter box or sweep up his hair from rugs.” In another study [30], the humanoid robot Alice was described as follows:

Very accurate. They will not be hasty, or they will not have a bad mood.…They won’t forget anything.…They will not be distracted, will not have a headache. I think it is positive that they will always be 100%.…Available.…Concentrated.…Trustworthy.

In addition, other emphasized aspects were features like a nice physical appearance [93,97], inviting and “unintimidating” attitudes and cuteness [97], and relational support [115].

These results, however, were mirrored by criticism. While some older adults appreciated SARs for their “roboticism” and “nonsimilarity” to living beings, others despised the fact that they are programmed to “pretend” to be what they are not. Indeed, some older adults refused their inauthenticity and lack of spontaneity [30,92,94,95,100]. Their simulation of living beings’ capabilities (affective, social, etc) was strongly rejected as phony. For example, in 1 study [94], ElliQ, a nonmobile robot, and Vector, a toy-like robot, were described as follows:

…lacking feeling and being “cold.”…Stephanie said: …I don’t’ like the sort of seeming humanizing of the whole thing. It’s ridiculous.

Even Biscuit [94], which was praised for its “dog-like affection” by 1 older adult, was criticized by another:

…the simulation of a living dog and the reactions it prompts are phony.…A live dog is “not programmed to be joyous when it sees you” (Sarah).…Gwen…found Biscuit repellent because, “It’s false. It’s something that I’m trying to be happy or I’m trying to be something that I’m not.”

This criticism was reiterated in another study [95], where an older adult said that “the only thing that a robot could do to me or respond to me would be a response that’s been built in, it’s not spontaneous.” The critique of the lack of authenticity was connected to the rejection of the robot-provided mode of care, as stated in a study [30], in response to the use of Alice:

…only could interact in a preprogrammed manner.…According to most participants the care SARs provide to older adults would lack a fundamental quality of human care: “empathy,” “care for the hearth” and so forth.

In 2 papers [71,91], the issue of human-robot interaction emerged in connection with the use of CMTs and UNMTs. In particular, older people complained about a sense of anxiety and doubt. In the first study [71], talking about some digital services, such as Amazon and Google, activity trackers like Fitbit, and home energy sensors like Nest, a participant said:

Although I use the computer, I do find it quite frightening. The reason is that I don’t understand it. And I don’t know how to put things right.

In the second study [91], it was mentioned that telehealth technologies, such as videoconferencing, elicited anxiety for various reasons: “(1) receiving spam, (2) experiencing system updates, (3) losing written text, (4) damaging a device, (5) fearing the use of technology in general, (6) fearing microwave radiation, (7) fearing inadequate privacy protection, (8) feeling unsafe using the internet, and (9) fearing online scams or cyber criminals.”

### PHV Dimension

PHV was reported in 36 out of 70 publications, thus constituting the third most frequently cited dimension by participants in qualitative studies. An analysis of these 36 studies showed that in most cases, ETs appeared to have a positive impact on PHV.

Indeed, 33 studies revealed that ETs mitigated PHV, which was interpreted as nonpathological physical or physiological bodily deterioration related to aging. Participants appreciated ETs, particularly UNMTs, for different reasons. ETs with monitoring functions can detect emergencies like accidents and falls [30,37,46,58,62,64,66,75,79,81,87,99,100,116] and can monitor health conditions, daily habits, therapy compliance, and health changes [54,56,57,70,75,77,81,83,85,99,101,104]. They were also useful in some cases to monitor the environment and the opening of doors and windows [61,65,70,74,92,100]. These factors, according to older adults, increased the sense of safety and security [37,46,55,65,66,70,72,74,75,77,79,81,83,85,92,100,101,104]. This positive impact was associated with the possibility of remaining in one’s own home and avoiding institutionalization in a nursing home [66,77,83,101]. For example, in 1 study [75], the importance of environmental sensors for both fall detection and detection of physical decline was emphasized:

Mrs. D explained this as follows: “Well, you are on your own, so something can happen, like when you fall and can‘t get up.…I had the idea that this should be watched by someone somewhere.”…Mrs. A expressed this as follows: “if there should be a slow change in my daily pattern, I certainly wouldn’t report it. I wouldn’t notice, and therefore, I find it important that the nurse’s station gets a signal like: keep an eye on that”

Similarly, in 1 study [100], the robot Jibo was admired for the home security it provides:

P12 told a story of how the robot could help them…“It seems like a useful idea that if I’m sleeping, and I could have a robot that detects something unusual that I would like to be alerted to…You could program the thing and say if I have a sound like somebody trying to get through the window, please wake me up.”

Other participants highlighted how the use of ETs, above all wearable activity trackers, such as Garmin Vivofit 2, Fitbit, Apple Watch, Jawbone, Misfit Nike, and Gear Fit, which automatically track and monitor various indicators of physical activity (eg, steps taken, pulse or heart rate, calories consumed, etc), could motivate them to be more physically active and improve their health status [30,57,75,82,84,86]. For instance, in 1 study [86], it was stated:

Participants agreed that tracker use motivated them to walk more, driven by quantifying activity (counting steps) and continuously making users “more conscious of extra walking” (female participant, non-user).

Finally, the last important positive aspect concerning PHV in terms of nonpathological deterioration was the physical support and assistance SARs can provide [99,115], assisting older adults in house chores and picking up and transporting objects.

Some studies [30,58,61,77,81,88,117] showed that ETs can also mitigate PHV linked to pathological physical age-related conditions. Some participants perceived ETs, mostly UNMTs, as useful tools to prevent diseases and manage illnesses, such as diabetes, heart failure, dementia, and Alzheimer disease [58,77,81,88,117], and physical impairments, such as vision deficits [30,61]. In 2 studies [79,91], however, participants reported that ETs could exacerbate PHV. They were concerned about the possibility of CMTs and UNMTs negatively impacting a person’s health, causing diseases (eg, cancer). For example, in 1 study [79], a participant asked:

Could the sensor radio waves give you cancer? I think this is what I would be worried about.

In another study [91], the use of telehealth technologies, such as videoconferencing, elicited similar comments, and older adults expressed their fear of microwave radiation.

### PV Dimension

PV was the fourth most frequently mentioned dimension by older adults, appearing in 33 out of 70 studies. These 33 papers dealt only with strictly psychological dimensions (cognitive, emotional, or both).

The emotional aspects were those most often mentioned by older people, and they appeared in 23 out of 33 publications. Of these studies, 21 showed how ETs could mitigate PV. In only 8 studies, older adults highlighted the negative aspects.

Focusing on the positive comments, according to participants, the use of ETs could have a positive influence on older adults’ emotions and improve their mood: CMTs and UNMTs could provide a sense of safety, security, and reassurance [37,61-64,69,74,80,101,107]. With their monitoring functions, these devices not only contribute to improving PHV but also provide peace of mind. For example, in 1 study [62], a participant commented on the use of smart home technologies, saying that “his friend has decided to use such a device to have ‘peace of mind.’” ETs in general, but especially SARs, are stimulating tools that older people enjoy using [46,82,86,92-94,97,112]. Hobbit [92], a robotic platform with personal and social functionalities, raised the following comments:

He‘s interesting and amusing.

It is great fun when he is working well.

When he moves and his head moves, I get happy and compassionate.…He’s charming.

Another SAR, PARO [69,110], was really appreciated because of its calming effect and its positive influence on emotions. The use of other technologies [46,86,106,112] conveyed a sense of achievement and appreciation. Finally, the appearance of the technology, especially in the case of robots that look like living creatures, aroused positive feelings in users (eg, empathy, compassion, and tenderness) [93-95].

On the other hand, 8 studies revealed how ETs, mostly UNMTs, could have a negative effect on PV, generating adverse emotions and negative attitudes, such as irritation, frustration and annoyance [68,94,95,106], fear [55,56,107], reduced sense of safety [37], and self-doubt and lack of self-worth [106]. For instance, the robot ElliQ was criticized for its cold manners and its disturbing intrusiveness. Participants said:

It talks all the time.

I don’t know whether that would drive me mental if it kept interrupting me and telling me what to do…I might want to get an ax and cut it up.

In 1 study [107], older adults, reflecting on the use of UNMTs and CMTs, stated that they evoked the fear of “not being able to manage the technology, making a fool of oneself, and having to ask for help.” Moreover, the following statement was made: “Also, fear of being forced into something unwanted and of ruining something was described.” Finally, another study [106] stated that even the use of more common technologies, such as tablets, smartphones, and laptops, has challenged older adults, who feel incompetent when they are unable to use the devices, experiencing a sense of inadequacy:

Jennifer (female, 71, tablet) demonstrate how her frustration with online activity leads to self-doubt: …“Is it me doing something wrong?”

With regard to the cognitive level, 8 studies [30,57,67,82,99,102,108,118] showed how ETs could have a positive effect, compensating for memory deficits through the reminder function [30,57,67,99,102,108], influencing motivation and awareness [82], or improving intellectual functions [118]. For example, in 1 study [67] analyzing CMTs and UNMTs like smartwatches, an older adult said:

I think it’s good because there’s some people who as time goes by lose certain of their faculties as time goes by, and memory beginning to fade and so on.

In another study [82], the Jawbone bracelet, an UNMT that monitors daily activity, was described as follows:

I was motivated by the technology, that I freely admit.…I have walked a little more while being monitored.

In another study [118], participants said:

It [the tablet] keeps the brain active.

Learning any new skill, surely is helping the cognitive function.

At the same time, paradoxically, 5 studies [56,76,95,113,118] revealed that ETs could have a negative impact on the cognitive aspects of PV, because they could make older adults more aware of their cognitive decline [56,113] or they could cause a loss of intellectual functioning [76,95,118].

### SPEV Dimension

SPEV appeared in 25 publications. According to 1 study [38], sociocultural vulnerability (SCV) arises when older adults experience social exclusion, isolation, marginalization, or stigma, exacerbated by gender discrimination, educational disparities, and low social support. Economic vulnerability (EV) and political vulnerability (POV) include discrimination in health care access and exposure to unjust judicial systems. These 3 SPEV subdimensions will be analyzed separately.

SCV was identified in 13 studies. Most of these focused on the isolation of older adults and their exclusion from social life, highlighting the positive [58,80,104,106,109,111,112,115] or negative [72,77,101,118] impact of ETs (CMTs and UNMTs for the most part) on SCV.

In 8 studies addressing the advantages of ETs, older adults said that these devices can help them integrate into society and reduce the sense of isolation. For example, in 1 study [115], the SAR was appreciated because it could provide company for older adults, in addition to performing house chores: “Robots will be welcome if I can return to my home, where they can bring me water and live and play with me… (Male, participant 13).” CMTs, such as tablets (eg, iPad) [112], were seen as tools to stay connected to not only family and friends, mitigating RV, but also society and the outside world at large:

Harold stated: “I feel more informed; I feel I‘m in more contact with my family.”…Connie said, “I feel like I’m connected to the world.”…“Well, I feel like I‘ve come up in the world.”…Carol, 80-years-old, said she felt more “modern…I’m up par with other people.” Mary, who is 89-years-old, mentioned that she felt like she was “coming into the 21st century.”

However, some studies indicated that ETs may exacerbate SCV by increasing isolation and social exclusion. Specifically, older adults expressed concerns about losing human contact, abandonment, and isolation linked to the growing use of monitoring technologies [72,77]. In 1 study [77], participants said that ambient assisted living technologies “should not replace human care…‘Nothing can replace human contact. The more helpless you are, the more you need for people to come and check on you once in a while.’” In another study [101], the use of CMTs (ie, assistive technology services) made older adults fear being “neglected by the official health care system and society.” In another study [118], the concern was different, as the cause of isolation was not the excessive use of monitoring technology, but rather older adults’ lack of access to it or inability to use it, which prevented them from keeping in touch with others and the society as a whole:

“I’d really like to be in the modern world and to be able to manage these things and to be able to access more. I just feel very limited in what I’m doing.…” (G2, P6). Participants in the no computer experience group also stated: G3, P6: “I think we’re missing out a lot, because all the information is at hand and we don’t know how to collect that information.”…G3, P5: “I just think we’re like a forgotten generation, that’s what I feel like. You want to go in and you want to be able to talk with your family and your grandchildren and not look vacant when they say, I’m going to do this.”

In 2 studies, older people focused on the barriers to ET use. Participants in the first study [104] expressed concerns about access to CMTs and about their inexperience with these devices. In the second study [114], older adults stressed how different understanding and use of technology between young and older people expose intergenerational differences, and the paper stated:

Grandchildren preferred to send videos and receive video/images, while participants mostly sent audio and preferred to receive text messages. Additionally, grandchildren would not receive replies within a day and thought it was frustrating. Grandparents showed surprise and confusion when we told them that grandchildren were expecting quicker replies, as they wanted to “take time and think about what to say back.”

A study on home monitoring techniques (wireless motion sensors, in-kitchen temperature sensors, door contact sensors, and pressure mats) considered SCV by examining older adults’ living situations [58]. It highlighted the positive contribution of ETs in reducing abandonment [58], as these technologies compensate for the lack of support from children.

EV was addressed in 13 studies. All of them, except for 1 study [89], highlighted how ETs exacerbate this vulnerability. Most older adults’ comments indicated that ETs have a strong financial impact on their income. Many participants expressed concerns about the high costs of these devices and their maintenance, which they could not afford [30,57,62,65,66,72,79,83,88,90,105,118]. This criticality emerged for all 3 types of ETs, but especially in relation to UNMTs, such as smart home technologies. Only 1 study reported how mHealth apps mitigate EV, as they could allow older adults to save money on health management [89]:

I mean one of the things I think is terrific that we can email our doctors and get an answer quickly. I’m all for it. It makes good sense. Participant 11.

Finally, POV was discussed in only 1 study [111], and older adults found that the use of CMTs had a negative impact on this vulnerability. The study showed that ETs exacerbate POV because the lack of skills and knowledge on ETs could complicate older adults’ participation in political life and decrease their possibility to be informed and obtain economic benefits [111].

### ESV Dimension

Lastly, ESV was by far the least cited, as it appeared in only 1 publication [113]. In this study [113], according to the participants, a CMT, such as a tablet-based communication app, which allows asynchronous multimedia communication, can exacerbate ESV because it makes older adults aware of their existential limitations and of their finitude:

They reported how “inadequate” and “limited” they felt. For Ike (aged 74), the technology made his “Parkinson’s battles” more noticeable, from eyesight problems to “losing cognitive abilities” when he forgot about some app’s “features.” The technology emphasized his health status and a compromised sense of personhood and identity: “I was not like this before,” he told us.

### Usability

Beyond the ethical impact of ETs on older adults’ vulnerabilities examined through a top-down approach, adopting a bottom-up perspective revealed significant findings regarding usability. Usability issues were addressed in 36 of the 70 publications reviewed. According to the International Organization for Standardization (ISO), usability is “the extent to which a system, product, or service can be used by specified users to achieve specified goals with effectiveness, efficiency, and satisfaction in a specified context of use” (ISO 9241-11:2018) [119]. Usability was reported to significantly shape users’ perceptions of a technology’s usefulness and acceptability [120].

Regarding this topic, we observed that participants’ perceptions related to the usability of ETs could be both positive and negative. A total of 18 publications highlighted the advantages of all types of ETs (CMTs, UNMTs, and SARs). However, critical issues were emphasized more often in 25 publications.

The first usability issue identified was the design or the interface of the devices, which raised many concerns among older adults. It was described by participants as uncomfortable or cumbersome [47,61,69,70,80,86,105,114,116,118]. For example, in 1 study [69], with regard to UNMTs (ie, motion and door sensors, smoke detectors, and fall detectors), the following information was provided:

Several older adults remarked on the light flashes coming from sensors.…She did not see its benefits and was also bothered by the light emitted from the sensors: “The watch kept falling out of her hand. It bothered her when she slept. She asked why the sensor light was flashing, why it was red, why this, and why that. It really drove me crazy. Then she said she did not want to wear it anymore, that it was useless” (Carer 5).

Similarly, in another study [86], wearable activity trackers, in particular Garmin Vivofit2, elicited the following critique:

Nonusers described the band as plastic (ie, cheap and of bad quality), clunky, annoying, rigid, and uncomfortable: “It’s very rigid. The design is poor. It collects water underneath. I end up having a really loose bracelet. Which could have some effect on accuracy. I don’t know. I found it totally uncomfortable. It’s really ugly.”

With regard to SARs, in 1 study [92], Hobbit was deemed “too big,” and it was mentioned that “he gets very hot” and generates too much noise. In another study [116], the concern was about the excessive bulkiness of SARs, which could be dangerous if the older person lives in a small house with “potential presence of architectonic barriers.” Moreover, the following statement was made: “Older adults’ worries were also expressed in relation to the furniture or ornaments located inside their houses that could be damaged by robot movements.” Other critical remarks were made about ETs, which were not user-friendly [84,91,106,121] and hard to understand [70,71,79,84,88,89,95]. In 1 study [84], older adults showed reluctance to use the wearable device Fitbit, which can monitor physical activity and vital signs:

…doubted their ability to manage the “complex” system.…For example, one senior said that “It sure takes a long time to use this Fitbit, I’m not sure it’s worth it.”…After using the devices,…seniors were more concerned with the output complexity of the wearable device, ie, the complexity of the measurements provided, expressed in incomprehensible data for them.

Another type of UNMT, an integrated smart home system [70], was criticized in similar terms:

One participant…perceived the usability of an ISHS very complicated and was very hesitant to continue using an ISHS, “I find it difficult to use this technology, so I am not very confident I could properly use them in my life.”

Moreover, the interaction with technology evoked in the older adults feelings of frustration [29,70,82,84,89,90,95,99,111], disappointment [86], doubts about their efficacy and anxiety [82,86,91], fear [80], and apprehension [79]. These feelings sometimes emerged because of the device’s inaccuracy and/or errors [84,86,99].

Participants also complained about their lack of knowledge and experience with the technologies and mentioned that this is a barrier preventing them from using the technologies [29,82,88,89,118]. For example, in 1 study [88], interviewees believed that “not knowing how to operate” mHealth technologies, such as mobile phones, tablet computers, patient monitoring devices, and mobile apps, “kept older adults from using such technology.” Some interviewees commented:

I think they’re good, but I just don’t know how to use, to work it. I just don‘t have the knowledge on how to work it.

I think the first thing is the lack of technical know-how is one of the barriers.

Another barrier posed by some kinds of ETs was their inaccessibility to users with disabilities, such as reduced visual acuity [105].

Lastly, participants reported a sense of annoyance because the technology use was dependent on power supply and the need to charge the battery (and not simply replace it with a new one) [70,91,106].

Shifting the focus to publications that reported the benefits of ETs in terms of usability, many participants judged the different types of ETs as easy to use, user-friendly [46,54,61,75,82,87,88,92,98,109,112,114], and intuitive to operate and understand [57,87,107]. In other cases, the technology was appreciated because it was comfortable [70,90], versatile [118], and accessible, as it was designed with and for older people [114]. One paper showed how participants appreciated the communication service of SARs, enjoying the cooperation with the robots (ORO, DORO, and CORO) [93]. In 1 study [68], the reassuring features that indicated the technology was working were found to be important by older adults.

Analysis of several publications revealed that older adults have identified strategies to address 2 key usability issues: inadequate design/interface and lack of knowledge/experience. First, to improve the design of some technologies, participants were pragmatic in their recommendations [54,81,87]. For example, they asked for simple instructions, fewer buttons, larger fonts, manageable sizes, and speech-activated tools [81]. Second, to compensate for the lack of knowledge about technology, some participants suggested adequate and practical training on how to use it [79,88], while others highlighted the importance of support, encouragement, and education, considering that older adults need slow and individual guidance while using technology [111]. Other participants suggested receiving tailored instructions through readable and understandable manuals or handouts that accompany technology devices [79,81,118].

### Means to Address Vulnerabilities

Our analysis showed that older adults’ suggestions for addressing vulnerability were closely tied to the specific technologies examined. Four publications [66,79,88,113] described strategies to mitigate particular vulnerability dimensions, including PHV [79]; RV [113]; MV understood as privacy/confidentiality [66] and decisional autonomy/self-determination [66,79,113]; SCV, particularly regarding isolation/exclusion from social life [113]; and EV [88].

In 1 study [79], which involved UNMTs (wireless sensor network-based systems that collect a range of environmental and structural sensory information such as weight and blood sugar), participants expressed 2 contrasting views. Some participants prioritized autonomy and independence, arguing that “users should be allowed to turn the system off when desired,” thereby emphasizing MV mitigation related to decision-making. Others prioritized safety, proposing time limits on deactivation or preventing users from turning the system off entirely, given its purpose of enabling prompt medical assistance. PHV containment took precedence, even at the cost of reducing individual autonomy.

In another study [66], which was also focused on UNMTs, older adults suggested mitigating privacy-related MV by “processing the information before it is transferred.” To further address MV interpreted as decisional autonomy, older adults emphasized that they “should decide what information is collected and transferred and to whom” and that technologies should be tailored to self-determined needs.

Regarding RV, participants in 1 study [113], which reflected on the use of a CMT (tablet-based communication app), stressed the need to “destigmatize loneliness, making it ‘OK to talk about it’ (Elsie, 86)” and avoid exacerbating reduced agency or compromised personhood. For this purpose, they recommended to “(1) understand interests and backgrounds to identify interventions, (2) provide a list of options for people to choose from and experiment with, and (3) ensure activities that entail active involvement and afford opportunities for meaningful interaction within and across generations.” These suggestions support decisional autonomy and call for the improvement of SCV, understood in a broad sense as the possibility of connection and interaction with others and specifically as the reduction of isolation and exclusion from social life, by fostering intergenerational relations.

Finally, in 1 study [88], to address EV, participants recommended providing mHealth tools free of charge:

I would consider this kind of technology only if it were given to me.

Well, I tell you like this, if they are going to pay for me to use one, then I will use one for my health…

## Discussion

### Main Findings

This review aimed to systematically examine the perceptions and experiences of healthy older adults exposed to CMTs, UNMTs, VRTs, and SARs, and to investigate the ethically related impact of such technologies on the vulnerabilities of these older adults.

MV emerged as the most frequent concern. Many older adults emphasized the potential negative impact of ETs, especially monitoring technologies (wearables and environmental sensors), on privacy in terms of confidentiality, data handling, and disclosure to third parties. Others noted positive implications for autonomy and self-determination, as these devices provide greater control over oneself and one’s living environment.

RV was identified as the second most frequently mentioned vulnerability. ETs positively influenced relationships among older adults, care staff, caregivers, and family members by supporting independence and community living while also facilitating practical and emotional support. Concerns remained that technologies might replace human interaction, reduce face-to-face care, increase misunderstanding and mistrust, or exacerbate loneliness, and this is consistent with prior literature, particularly regarding SARs [23,122-125].

Comparing these findings with those of another systematic review on older adults with cognitive impairments [126], we observed similar ambivalence. ETs can mitigate existing vulnerabilities but can also create new and/or worse (already existing) vulnerabilities. This depends on both the specific type of technology used and the dimension of vulnerability considered.

Both cognitively impaired older adults and healthy older adults recognized the positive impact of ETs on PHV, particularly through the ability of UNMTs to monitor health changes, detect falls and emergencies, and identify intruders, confirming previous findings [41].

A similar ambivalence emerged with RV. Yet, unlike cognitively impaired individuals (who focused on PV, RV, and PHV), cognitively healthy older adults emphasized the negative impact of ETs on MV. UNMTs, in particular, appeared to interfere with decisional autonomy, jeopardize privacy (confidentiality), compromise privacy through control and surveillance, undermine integrity and identity, and cause stigmatization and infantilization. This aligns with concerns described in the so-called *Big Brother Syndrome* [23], that is, the perception of being constantly observed by monitoring systems and feeling bewildered by the presence of numerous devices, which invade personal space [125]. In general, the literature shows that older adults are often willing to accept some privacy infringements in favor of safety and other benefits provided [127], but they nonetheless expect ETs to be reliable and trustworthy [128] and often react negatively to the invasiveness, intrusiveness, and obtrusiveness of ETs [122].

In conclusion, older adults with cognitive impairments tend to accept more tradeoffs in exchange for even modest physical or psychological benefits, due to greater psychophysical fragility, whereas cognitively healthy older adults tend to prioritize general, age-independent values, most notably privacy and autonomy.

### Strengths and Limitations

The main strength of this work is that our study design is unique in that there are no other systematic reviews examining the relationship among ETs (taken as a whole), cognitively healthy older adults, and ethically related content, with a focus on vulnerability. Second, this review allows both a broader overview of the impact of ETs on older adults’ vulnerabilities and a detailed analysis of how *specific types* of ETs affect *distinct* vulnerability dimensions. In other words, our research is highly informative with regard to both its *breadth* (ie, the number of ETs included) and *depth* (ie, the fine-grained scrutiny it enables on the specific challenges that every single family of ETs raises on distinct dimensions of older adults’ vulnerabilities). Third, as a complement to another systematic review of qualitative studies [126], this review contributes to advancing a broader research agenda on aging that includes both cognitively healthy and cognitively impaired populations. Finally, by reviewing qualitative studies conducted with older adults, this research foregrounds their perspectives, including needs, desires, and concerns.

There are some limitations. First, the search was conducted 2 years ago and included only English-language articles, potentially limiting its temporal and linguistic scope. Second, some included studies may have inadvertently involved participants with cognitive disorders (eg, Alzheimer disease), as screening for such conditions was not always explicitly reported in the primary research. Third, as this is a systematic review rather than a trial, the findings are not generalizable. Nonetheless, by synthesizing all available translated studies on the topic, this work offers a broad overview and highlights the conceptual trends of potential relevance.

### Conclusions and Future Directions

This study offers a solid theoretical foundation for future research, especially on strategies for addressing vulnerability in the context of aged care. Understanding older adults’ vulnerabilities, especially in relation to ETs, is essential for identifying areas that demand greater sensitivity and tailored care approaches. Privacy and data confidentiality concerns (risks of breaches and data misuse or abuse) are crucial when deploying monitoring technologies, especially those involving audio or video recording in intimate settings. Cost also remains a significant barrier. Many older adults express interest in adopting ETs, but financial constraints may hinder their ability to do so. Equally important are the psychological impacts that such technologies may provoke, including anxiety, frustration, irritation, and feelings of inadequacy. Both ethics-by-design solutions and psychosocial interventions may help mitigate these effects.

Future research would benefit from complementing this theoretical framework with empirical studies aimed at identifying domains where psychological, sociopolitical, and economic interventions are most needed. Qualitative methodologies would be particularly useful to capture the perspectives of key stakeholders, including formal and informal caregivers, health care professionals, and policymakers.

## Supplementary material

10.2196/69676 Multimedia Appendix 1 Groups of organizing concepts for searching the literature and their associated database search terms.

10.2196/69676 Multimedia Appendix 2 Search strings used for searching databases stratified by organizing concepts.

10.2196/69676 Multimedia Appendix 3 Inclusion and exclusion criteria.

10.2196/69676 Multimedia Appendix 4 List of included publications.

10.2196/69676 Multimedia Appendix 5 Detailed characteristics of the included publications.

10.2196/69676 Multimedia Appendix 6 Example of the Qualitative Analysis Guide of Leuven conceptual scheme.

10.2196/69676 Multimedia Appendix 7 Ethically related impact of emerging technologies on older adults’ vulnerabilities as gleaned from the perceptions and experiences of the older adults.

10.2196/69676 Multimedia Appendix 8 Quality assessment of the included articles (Critical Appraisal Skills Programme).

10.2196/69676 Checklist 1 PRISMA checklist.

## Abbreviations

CMT: conventional monitoring technique
ESV: existential or spiritual vulnerability
ET: emerging technology
EV: economic vulnerability
MV: moral vulnerability
PHV: physical vulnerability
POV: political vulnerability
PRISMA: Preferred Reporting Items for Systematic Reviews and Meta-Analyses
PV: psychological vulnerability
QUAGOL: Qualitative Analysis Guide of Leuven
RV: relational or interpersonal vulnerability
SAR: socially assistive robot
SCV: sociocultural vulnerability
SPEV: sociocultural, political, and economic vulnerability
UNMT: unconventional monitoring technique
VRT: virtual reality technique
