# Supplementary material for: Emerging Technologies and Vulnerabilities in Older Adults Without Cognitive Impairments: Systematic Review of Qualitative Evidence
Source: Interact J Med Res. 2026 Feb 19;15:e69676. doi: 10.2196/69676 (PMC12919910; doi:10.2196/69676)
Supplement: Multimedia Appendix 1 [file ijmr-v15-e69676-s001.docx]

**Multimedia Appendix 1.** Groups of organizing concepts for searching the literature and their associated database search terms.

| **Group 1: Population** | **Group 2: Technology** | **Group 3: Vulnerability** |
| --- | --- | --- |
| Aged; aging; elder; elderly; older people; older adult; old people; older person; old adult; aged care; geriatrics; older user | Assistive technologies; converging technologies; digital technologies; emerging technologies; health technologies; innovative technologies; new technologies; robotic technologies; telehealth technologies; robots; robotics; assistive robots; companion robots; domestic robots; social robots; social service robots; internet of things; domotics; smart home; wearables; wearable devices; ambient intelligence; information and communication technologies; care technologies; tracking devices; surveillance technologies; telecare; remote monitoring technologies; digital platforms; virtual reality | Vulnerability; frailty; fragility; frailness; acceptance; attitude; concerns; discomfort; distress; ethical issues; ethics |
