# Supplementary material for: Emerging Technologies and Vulnerabilities in Older Adults Without Cognitive Impairments: Systematic Review of Qualitative Evidence
Source: Interact J Med Res. 2026 Feb 19;15:e69676. doi: 10.2196/69676 (PMC12919910; doi:10.2196/69676)
Supplement: Multimedia Appendix 2 [file ijmr-v15-e69676-s002.docx]

**Multimedia Appendix 2.** Search strings used for searching databases stratified by organizing concepts.

| **Database** | **Group 1: population** |  | **Group 2: technology** |  | **Group 3: vulnerability** |
| --- | --- | --- | --- | --- | --- |
| Pubmed | "Dementia"[MeSH Terms:noexp] OR "Dementia"[Title/Abstract] OR ("Aged"[Title/Abstract] OR "Aging"[Title/Abstract] OR ("Aging"[MeSH Terms] OR "Aging"[All Fields] OR "ageing"[All Fields]) OR "elder*"[All Fields] OR ("Aged"[MeSH Terms] OR "Aged"[All Fields] OR "elderly"[All Fields] OR "elderlies"[All Fields] OR "elderly s"[All Fields] OR "elderlys"[All Fields]) OR (("older"[All Fields] OR "olders"[All Fields]) AND ("people s"[All Fields] OR "peopled"[All Fields] OR "peopling"[All Fields] OR "persons"[MeSH Terms] OR "persons"[All Fields] OR "people"[All Fields] OR "peoples"[All Fields])) OR (("older"[All Fields] OR "olders"[All Fields]) AND "adult*"[All Fields]) OR ("old"[All Fields] AND ("people s"[All Fields] OR "peopled"[All Fields] OR "peopling"[All Fields] OR "persons"[MeSH Terms] OR "persons"[All Fields] OR "people"[All Fields] OR "peoples"[All Fields])) OR "Aging"[MeSH Terms:noexp]) OR "Aged"[MeSH Terms] OR “Alzheimer Disease"[MeSH Terms] OR Alzheimer’s[Title/Abstract] | AND | "Self-Help Devices"[Mesh:NoExp] OR "Digital Technology"[Mesh:NoExp] OR "Biomedical Technology"[Mesh:NoExp] OR "Robotics"[Mesh:NoExp] OR "Internet of Things"[Mesh] OR "Ambient Intelligence"[Mesh] OR assistive technolog*[Title/Abstract] OR converging technolog*[Title/Abstract] OR digital technolog*[Title/Abstract] OR emerging technolog*[Title/Abstract] OR health technolog*[Title/Abstract] OR innovative technolog*[Title/Abstract] OR new technolog*[Title/Abstract] OR robotic technolog*[Title/Abstract] OR telehealth technolog*[Title/Abstract] OR robot*[Title/Abstract] OR robotics[Title/Abstract] OR assistive robot*[Title/Abstract] OR companion robot*[Title/Abstract] OR domestic robot*[Title/Abstract] OR social robot*[Title/Abstract] OR social service robot*[Title/Abstract] OR internet of things [Title/Abstract] OR domotics[Title/Abstract] OR smart home*[Title/Abstract] OR wearable*[Title/Abstract] OR wearable device*[Title/Abstract] OR ambient intelligence[Title/Abstract] OR information and communication technolog*[Title/Abstract] OR care technolog*[Title/Abstract] OR tracking device*[Title/Abstract] OR surveillance technolog*[Title/Abstract] OR telecare[Title/Abstract] OR remote monitoring technolog*[Title/Abstract] OR digital platform*[Title/Abstract] OR "Virtual Reality"[Mesh:NoExp] OR virtual reality[Title/Abstract] | AND | "vulnerability"[Title/Abstract] OR "vulnerab*"[Title/Abstract] OR "fragility"[Title/Abstract] OR "Frailty"[Title/Abstract] OR "frail"[Title/Abstract] OR "fragilit*"[Title/Abstract] OR "frailness"[Title/Abstract] OR "frailties"[Title/Abstract] OR "Frailty"[MeSH Terms] OR "Ethic*"[Title/Abstract] OR "Anxiety"[MeSH Terms:noexp] OR "anxiet*"[Title/Abstract] OR "acceptance"[Title/Abstract] OR "concern*"[Title/Abstract] OR "discomfort"[Title/Abstract] OR "distress"[Title/Abstract] OR "ethical issue*"[Title/Abstract] |
| **Number of results** | WITHOUT English Filter | 4.161 | WITH English filter | 4.041 |  |
| Web of Science | TS=(dementia OR "alzheimer disease" OR alzheimer* OR aged OR ageing OR aging OR elder* OR elderl* OR "older* peopl*" OR "older* person*" OR "older* adult*" OR "old peopl*" OR "old person*" ) | AND | TS=( "self help device*" OR "digital technolog*" OR "biomedical technolog*" OR robotic* OR "internet of things" OR "ambient intelligence" OR "assist* technolog*" OR "converging technolog*" OR "emerging technolog*" OR "health technolog*" OR "innovative technolog*" OR "new technolog*" OR "robotic technolog*" OR "telehealth technolog*" OR robot* OR "assist* robot*" OR "companion* robot*" OR "domestic robot*" OR "socia* robot*" OR "socia* service robot*" OR domotic* OR "smart home*" OR "information and communication technolog*" OR "care technolog*" OR "tracking device*" OR "surveillance technolog*" OR telecare OR "remote monitoring technolog*" OR "digital platform*" OR "virtual reality" ) | AND | TS=(vulnerabilit* OR fragilit* OR frailt* OR frail* OR ethic* OR "ethical issue*" OR anxiet* OR acceptance OR concern* OR discomfort OR distress) |
| **Number of results** | WITHOUT English Filter | 5.433 | WITH English Filter | 5.237 |  |
| Embase | dementia:ab,ti OR 'alzheimer s':ab,ti OR 'alzheimer disease':ab,ti OR aged:ab,ti OR aging:ab,ti OR ageing:ab,ti OR elder:ab,ti OR elders:ab,ti OR elderly:ab,ti OR elderlies:ab,ti OR 'elderly s':ab,ti OR elderlys:ab,ti OR 'older people s':ab,ti OR 'olders people s':ab,ti OR 'older peopled':ab,ti OR 'olders peopled':ab,ti OR 'older peopling':ab,ti OR 'olders peopling':ab,ti OR 'older persons':ab,ti OR 'olders persons':ab,ti OR 'older people':ab,ti OR 'olders people':ab,ti OR 'older peoples':ab,ti OR 'olders peoples':ab,ti OR 'older adult':ab,ti OR 'older adults':ab,ti OR 'olders adult':ab,ti OR 'olders adults':ab,ti OR 'old people s':ab,ti OR 'old peopled':ab,ti OR 'old peopling':ab,ti OR 'old persons':ab,ti OR 'old people':ab,ti OR 'old peoples':ab,ti | AND | 'self help device':ab,ti OR 'self help devices':ab,ti OR 'digital technology':ab,ti OR 'digital technologies':ab,ti OR 'biomedical technology':ab,ti OR 'biomedical technologies':ab,ti OR robotics:ab,ti OR 'internet of things':ab,ti OR 'ambient intelligence':ab,ti OR 'assistive technology':ab,ti OR 'assistive technologies':ab,ti OR 'converging technology':ab,ti OR 'converging technologies':ab,ti OR 'emerging technology':ab,ti OR 'emerging technologies':ab,ti OR 'health technology':ab,ti OR 'health technologies':ab,ti OR 'innovative technology':ab,ti OR 'innovative technologies':ab,ti OR 'new technology':ab,ti OR 'new technologies':ab,ti OR 'robotic technology':ab,ti OR 'robotic technologies':ab,ti OR 'telehealth technology':ab,ti OR 'telehealth technologies':ab,ti OR robot:ab,ti OR robots:ab,ti OR 'assistive robot':ab,ti OR 'assistive robots':ab,ti OR 'companion robot':ab,ti OR 'companion robots':ab,ti OR 'domestic robot':ab,ti OR 'domestic robots':ab,ti OR 'social robot':ab,ti OR 'social robots':ab,ti OR 'social service robot':ab,ti OR 'social service robots':ab,ti OR domotics:ab,ti OR ((('smart home':ab,ti OR 'smart homes':ab,ti OR wearable:ab,ti OR wearables:ab,ti OR 'wearable device':ab,ti OR 'wearable devices':ab,ti OR information:ab,ti) AND 'communication technology':ab,ti OR information:ab,ti) AND 'communication technologies':ab,ti) OR 'care technology':ab,ti OR 'care technologies':ab,ti OR 'tracking device':ab,ti OR 'tracking devices':ab,ti OR 'surveillance technology':ab,ti OR 'surveillance technologies':ab,ti OR telecare:ab,ti OR 'remote monitoring technology':ab,ti OR 'remote monitoring technologies':ab,ti OR 'digital platform':ab,ti OR 'digital platforms':ab,ti OR 'virtual reality':ab,ti | AND | vulnerability:ab,ti OR vulnerabilities:ab,ti OR fragility:ab,ti OR fragilities:ab,ti OR frailty:ab,ti OR frailties:ab,ti OR frailness:ab,ti OR frail:ab,ti OR ethic:ab,ti OR ethics:ab,ti OR 'ethical issue':ab,ti OR 'ethical issues':ab,ti OR anxiety:ab,ti OR anxieties:ab,ti OR acceptance:ab,ti OR concern:ab,ti OR concerns:ab,ti OR discomfort:ab,ti OR distress:ab,ti |
| **Number of results** | WITHOUT English Filter | 1.245 | WITH English Filter | 1.211 |  |
| CINAHL | "TI ( dementia or alzheimers or alzheimers disease ) OR AB ( dementia or alzheimers or alzheimers disease ) OR TI ( aged or ageing or aging or elder* or elderl* ) OR AB ( aged or ageing or aging or elder* or elderl* ) OR TI ( older* peopl* or older* adult* or older* person* or old peopl* or old person* ) OR AB ( older* peopl* or older* adult* or older* person* or old peopl* or old person* ) | AND | "TI ( self help device* OR digital technolog* OR biomedical technolog* OR robotics OR internet of things OR ambient intelligence ) OR AB ( self help device* OR digital technolog* OR biomedical technolog* OR robotics OR internet of things OR ambient intelligence ) OR TI ( assistive technolog* OR converging technolog* OR emerging technolog* OR health technolog* ) OR AB ( assistive technolog* OR converging technolog* OR emerging technolog* OR health technolog* ) OR TI ( innovative technolog* OR new technolog* OR robotic technolog* OR telehealth technolog* ) OR AB ( innovative technolog* OR new technolog* OR robotic technolog* OR telehealth technolog* ) OR TI ( robot* OR assistive robot* OR companion robot* OR domestic robot* OR social robot* OR social service robot* OR smart home* OR domotics OR wearable* OR wearable device* OR information AND communication technolog* OR care technolog* OR tracking device* OR remote monitoring technolog* OR surveillance technolog* OR digital platform* OR telecare OR virtual reality ) OR AB ( robot* OR assistive robot* OR companion robot* OR domestic robot* OR social robot* OR social service robot* OR smart home* OR domotics OR wearable* OR wearable device* OR information AND communication technolog* OR care technolog* OR tracking device* OR remote monitoring technolog* OR surveillance technolog* OR digital platform* OR telecare OR virtual reality ) | AND | TI ( vulnerabilit* OR fragilit* OR frailt* OR frail* OR ethic* OR ethical issue* OR anxiet* OR concern* OR acceptance OR discomfort OR distress ) OR AB ( vulnerabilit* OR fragilit* OR frailt* OR frail* OR ethic* OR ethical issue* OR anxiet* OR concern* OR acceptance OR discomfort OR distress ) |
| **Number of results** | WITHOUT English Filter | 1.085 | WITH English Filter | 1.053 |  |
| Philosopher’s Index | noft(dementia OR alzheimer* OR alzheimer* disease OR aged OR ageing OR aging ) OR noft(elder* OR elderl* OR older* peopl* OR older* adult* OR older* person* OR old peopl* OR old person*) | AND | noft(self help device* OR digital technolog* OR biomedical technolog* OR robotics OR internet of things OR ambient intelligence) OR noft(assistive technolog* OR converging technolog* OR emerging technolog* OR health technolog*) OR noft(innovative technolog* OR new technolog* OR robotic technolog* OR telehealth technolog*) OR noft(robot* OR assistive robot* OR companion robot* OR domestic robot* OR social robot* OR social service robot* OR smart home* OR domotics OR wearable* OR wearable device* OR information AND communication technolog* OR care technolog* OR tracking device* OR remote monitoring technolog* OR surveillance technolog* OR digital platform* OR telecare OR virtual reality) | AND | noft(vulnerabilit* OR fragilit* OR frailt* OR frail* OR ethic* OR ethical issue* OR anxiet* OR concern* OR acceptance OR discomfort OR distress) |
| **Number of results** | WITHOUT English Filter | 100 | WITH English Filter | 89 |  |
| **Total** | WITHOUT English Filter | 12.024 | WITHOUT English Filter | 11.631 |  |
