# Supplementary material for: Emerging Technologies and Vulnerabilities in Older Adults Without Cognitive Impairments: Systematic Review of Qualitative Evidence
Source: Interact J Med Res. 2026 Feb 19;15:e69676. doi: 10.2196/69676 (PMC12919910; doi:10.2196/69676)
Supplement: Multimedia Appendix 3 [file ijmr-v15-e69676-s003.docx]

**Multimedia Appendix 3.** Inclusion and exclusion criteria.

|  | **INCLUDED** | **EXCLUDED** |
| --- | --- | --- |
| **Types** | The publication is considered to be reporting on primary, empirical research using a qualitative or mixed-method approach. | Editorials, book chapters, position papers, guidelines, reviews, protocols, ethics policies, and ethics codes, theoretical works of argument-based literature (i.e., an article using ethical concepts derived from current or traditional ethical theories in order to  argue for a position or conclusion). |
| **Topic** | Use of the concept of vulnerability and/or related concepts (i.e., frailty, frailness, fragility), in relation to ET. | The concept of vulnerability and/or related concepts is not significantly used and/or is not used in relation to ET and/or it is used in a purely medical sense. |
| **Population and research domain** | Application to the specific field of aged care (broadly understood), with a specific focus on older adults without cognitive impairments, older than 65 years. | Reference to fields other than aged care (e.g., research ethics), and/or target population not represented by older adults without cognitive impairments older than 65years. |
| **Language** | The publication must be in English. | Publications in languages other than English. |
