# Supplementary material for: Emerging Technologies and Vulnerabilities in Older Adults Without Cognitive Impairments: Systematic Review of Qualitative Evidence
Source: Interact J Med Res. 2026 Feb 19;15:e69676. doi: 10.2196/69676 (PMC12919910; doi:10.2196/69676)
Supplement: Multimedia Appendix 4 [file ijmr-v15-e69676-s004.docx]

**Multimedia Appendix 4.** List of included publications.

| **Reference No.** | **First Author and Publication Year** | **Country of Affiliation** | **Aim** | **Design/methodology** | **Participants** | **ET** |
| --- | --- | --- | --- | --- | --- | --- |
| **1** | Akerlind Charlotta (2018) | Sweden | To explore how older adults (OA ^a^) and their relatives understand and experience safety in everyday life, in relation to ET | Qualitative design, using semi-structured interviews  Qualitative content analysis | 12 OA + 8 relatives  OA’s age: 71-93, both sexes, without cognitive impairments | CMT/UNMT: eHomecare  Four tools: a night camera for supervision at night, a videophone and portable videophone for daily check-ups, social interactions and reminders and electronic mailbox for reminders and information by SMS and MMS messages and emails |
| **2** | Bajones Markus (2020) | Austria | To explore how OA interact with the robot in their private homes | Mix method approach, containing in-depth Interviews [and a questionnaire] | 16 OA  OA’s age: 75 – 89, both sexes, without cognitive impairments | SAR: Hobbit Robotic platform  An assistive robot that offers useful personal and social functionalities to enable independent living at home for OA |
| **3** | Baric Vedrana (2019) | Sweden | To explore community-dwelling OA’s experiences learning and using ET | Qualitative design using focus group interviews  Content analysis | 20 OA  OA’s age: 66-85, both sexes, without cognitive impairments | CMT: RemindMe  It is an interactive digital calendar with mobile phone reminders.  It has three functions: (i) scheduling of activities/events, and reminders; (ii) active conformation of reminders sent by SMS; and (iii) a unique feedback system that registers self-monitoring information based on the user’s interaction with the system |
| **4** | Bevilacqua Roberta (2021) | Italy | To investigate the psychosocial determinants that lead to the acceptability and willingness to interact with SAR.  To provide strategies to support OA/robot interactions, analysing the nonverbal behaviour, emotional expression, and verbal communication of OA | Mix-method approach, using observation to collect quantitative and qualitative data  Video interaction analysis | 35 OA  OA’s age: 67-80, both sexes, without cognitive impairments | SAR: the Robot Era  It involves three robotic agents: the domestic robot (DORO), which operates at home; condominium robot, which works in the common areas of buildings (CORO); and outdoor robot (ORO), which moves in the streets |
| **5** | Bian Chao (2021) | Canada | To understand OA’s perceptions and preferences in ET that can be used to measure frailty criteria in home settings | Qualitative approach using focus group interviews  Inductive thematic analysis | 15 OA  OA’s age: 65+, without cognitive impairments | UNMT: 10 different types of technologies  These include smartwatches, chair and bed sensors, motion sensors, standard cameras, depth camera etc. |
| **6** | Boström Martina (2013) | Sweden | To describe how OA perceive ET in terms of personal privacy | Qualitative, descriptive approach using focus group interviews  Qualitative content analysis | 45 OA  OA’s age: 65+, both sexes, without cognitive impairments | UNMT: monitoring techniques.  These include sensors embedded in watches and clothing, which control blood pressure, heart rate and body temperature, the refrigerator door opening, and OA’ movements in the home |
| **7** | Broadbent Elizabeth (2012) | New Zealand | To investigate attitudes and preferences of OA, their relatives, and staff at a retirement village towards the use of robots in the village | A mix-method approach using focus groups [and questionnaires] | 7 OA and caregivers, staff/managers  OA’s age: 73-89, all females, without cognitive impairments | SAR: Health-care robots |
| **8** | Cabrita Miriam (2019) | The Netherlands | To investigate the attitudes of OA living independently regarding ET to support healthy behaviours, addressing nutrition, physical and cognitive function, and well-being | Qualitative approach using semi-structured interviews  Thematic analysis | 12 OA  OA’s age: 65-78  both sexes, without cognitive impairments | CMT/UNMT: Ambulatory technology.  It consists of a mobile phone, a Fitbit Zip step counter and a smart scale Withings 30. It can provide continuous real-time information on the health status of the individual, detect changes over time, and promote healthy behaviours to prevent or early detect decline |
| **9** | Cajita Maan Isabella (2018) | USA | To investigate potential facilitators of and barriers to the use of mobile and/or wireless devices (mHealth) among OA | Qualitative approach using semi-structured interviews  Qualitative content analysis | 10 OA  OA’s age: 66-83, both sexes, without cognitive impairments | CMT/UNMT: mHealth technologies  It consists of mobile and wireless devices, such as mobile phones, tablet computers, patient monitoring devices, and mobile applications (apps).  These tools are used to support the achievement of health objectives |
| **10** | Chang Chi-Ping (2017) | Taiwan | To explore OA’s perceptions and suggestions regarding using telehealth for diabetes management | Qualitative approach using semi-structured interviews  Thematic analysis | 18 OA  OA’s age:65-91, both sexes, without cognitive impairments | UNMT: Telehealth service  It integrates healthcare with informatics and involves the transfer and exchange of health information through electronic devices.  A home telehealth service also involves the remote delivery of health-related services via information and communication technologies (ICT) between a patient and a healthcare professional |
| **11** | Choi Yong K. (2021) | USA | To explore OA’s perceptions of Internet-of-Things (IoT) smart home devices and to describe what factors affect adoption of these technologies | Qualitative approach using semi-structured interviews  Thematic content analysis | 37 OA  OA’s age: 65+, without cognitive impairments | UNMT: Smart Home and IoT  A smart home is a residence that integrates technology within the home to enhance residents’ living by monitoring and supporting their health and wellness. IoT is the network of everyday objects equipped in internet connectivity.  These include a door/window sensor, a multi-sensor, a smart speaker and an IP (Internet Protocol) web camera |
| **12** | Chung Jane (2017) | USA | To explore OAs’ attitudes toward and perceptions of home-based monitoring technologies in a cultural context | Qualitative approach using focus groups and interviews  Thematic analysis | 21 OA, Korean Americans, and Koreans  OA’s age: 65 +, without cognitive impairments | UNMT: Home monitoring techniques  These include but are not limited to (1) activity monitoring that employs wireless motion sensors, in-kitchen temperature sensors, door contact sensors, and/or pressure mats; (2) sleep monitoring that detects postural changes or cardiorespiratory function; and (3) safety monitoring that detects falls |
| **13** | Coghlan Simon (2021) | Australia | To explore attitudes and perspectives of OA living independently but alone in their own homes, about three companion robots | Qualitative approach using semi-structured interviews  Thematic analysis | 16 OA  OA’s age: 65+, both sexes, without cognitive impairments | SAR: three companion robots.  These are ElliQ, a non-mobile home assistant robot; Vector, a toy-like robot; Biscuit, an animal-like robot.  ElliQ can display pictures and videos, has a head that can move around flexibly and lights up in the center when interacting. It has conversation capabilities and a female-sounding voice.  Vector can autonomously move around in its environment. It alters its behaviour with interaction. It can use speech to answer question and respond to spoken commands.  Biscuit is a non-mobile smallish dog, with a relatively lifelike appearance; it can respond to touch and speech by moving and making dog-like vocalizations. |
| **14** | Courtney Karen L. (2008) | USA | To explore OA’s privacy concerns and the willingness to adopt smart home Information Technologies (IT) within a residential care living environment | Descriptive qualitative approach using focus groups and interviews  Grounded theory analytic approach | 11 OA  OA’s age: 65+, both sexes, without cognitive impairments | UNMT: Smart Home IT  These tools are information-based technologies that collect and share information with the resident and their health care providers, in order to help individuals with tasks they would otherwise be unable to do or to help individuals to perform tasks more easily or safely |
| **15** | As above (2008) | USA | To explore the factors that influence the willingness of OA living in independent and assisted living continuing care retirement communities to adopt smart home technology | Descriptive qualitative approach using focus groups and interviews  Grounded theory analytic approach | 14 OA  OA’s age: 65+, without cognitive impairments | UNMT: Smart Home Technologies  These devices are information-based technologies that passively collect and share resident information with the resident and family members in addition to primary care providers.  These tools collect multiple types of data (e.g., physiological, location or movement data). Algorithms transform the raw data in activity patterns which can be used for early detection and intervention |
| **16** | Daniele Katia (2019) | Italy | To explore the needs, preferences, and views on ICT of some prefrail OA living alone  To explore their attitude toward a hypothetical caregiver robot and the functions they would ask for | Qualitative approach using semi-structured interviews  Interpretative phenomenological analysis | 25 OA  OA’s age: 65+, both sexes, without cognitive impairments | UNMT: ICT-based home services  SAR: Giraff, a robot implemented in the MoveCare project |
| **17** | Davenport Rick D. (2007) | USA | To explore the initial reactions of an elder concerning a prototype technology in a smart house | Mix method approach using an interview [and a questionnaire] | A 78-year-old elder woman without cognitive impairments but a right shoulder pain with limited right shoulder elevation of 90◦ | UNMT: Gator Tech Smart House  It includes different applications such as voice activation, smart front door, smart floor tracking system, security system, smart wave |
| **18** | Demiris George (2004) | USA | To investigate OA’s perceptions and expectations about ET installed and operated in their homes with the purpose of improving their quality of life and/or monitoring their health status | Qualitative approach using focus groups | 15 OA  OA’s age: 65+, without cognitive impairments | UNMT: Smart Home  It is a residence equipped with technology that can enhance safety of OA at home and monitor their health conditions |
| **19** | As above (2008) | USA | To present a participatory evaluation developed and tested with an actual smart home project implemented in a real-world setting | Qualitative approach using observations and interviews | 9 OA  OA’s age: +65, without cognitive impairments | UNMT: In-Home Monitoring System (IMS).  IMS consists of a set of wireless infrared proximity sensors to detect motion and pressure switch pads (sensor mats), a stove temperature sensor, sensors on cabinet doors and a bed sensor capable of detecting presence, respiration, pulse and movement in the bed. The Data Manager collects data from the sensors, date-time stamps the data and logs it into a file that is sent to a secure server as binary streams stripped of identifiers |
| **20** | As above (2008) | USA | To investigate OA’s perceptions of specific smart home technologies used by the Tiger Place project; perceived advantages and concerns associated with these types of technology; willingness to adopt such technologies in their residence; and preferences about recipients of sensor-generated information about their activity levels, sleep patterns, and potential emergencies | Qualitative approach using focus groups  Data driven content analysis | 14 OA  OA’s age: 65+, both sexes, without cognitive impairments | UNMT: Smart Home  It is a residence equipped with technology that can enhance safety of patients at home and monitor their health conditions.  It includes a bed sensor, gait monitor, stove sensor, motion sensor, and video sensor |
| **21** | Dermody Gordana (2021) | Australia | To explore the factors that influence community-dwelling OA’s readiness to adopt smart home technology to help guide the practical development and utilization of these technologies to meet the OA’s needs | Qualitative approach using focus groups and semi-structured interviews  Thematic analysis | 19 OA  OA’s age: 68-87, without cognitive impairments | UNMT: Smart Home  It is a technology that is designed to assist older adults with chronic disease in managing their health and to help extending independence to facilitate living in their own home for as long as possible |
| **22** | Deutsch Inbal (2019) | Israel | To provide a comprehensive mapping of healthy OA’s attitudes and concerns about some robots, each manifesting a different design approach | Qualitative approach using interviews  Thematic analysis | 30 OA  OA’s age: 67-90 (10 males and 20 females), without cognitive impairments | SAR: Home Robotic Devices  These include the following.  PR2 is a human-sized robot used by universities as a research platform. It is considered a prototypical example of a general home assistant robot.  Nao is a general purpose commercial anthropomorphic robot.  PARO is a robotic baby seal which is designed to interact with people, reacting to touch, light, audio, temperature, and posture changes.  ElliQ is a near-future commercial robot that has two parts: a screen and a ‘robotic character’.  Google Home is a commercially available smart device serving as a home assistant with a voice-activated speaker.  Cozmo is a small commercially available entertainment robot that can play games with users |
| **23** | Ehn Maria (2018) | Sweden | To explore OA’s experiences in using the current Activity Monitors (AM) and to learn more about OA’s requirements and preferences related to motivation, meaningfulness, usefulness, and usability | Descriptive qualitative study using Interviews  Inductive qualitative analysis | 8 OA  OA’s age: 75 -90, both sexes, without cognitive impairments | UNMT: AM and Tablet-based Apps  The AM are two commercially available bracelets for monitoring physical activity: Withings Activité Pop and Jawbone UP3, together with corresponding software (apps) accessible on a tablet.  The Withings bracelet is an analog wristwatch, displaying current time and giving feedback in terms of percentage of daily activity goal achieved. The Jawbone bracelet has three icons that can be lit up. It is used to monitor activity results |
| **24** | As above (2019) | Sweden | To investigate OA’s and health care professionals’ (HCPs) perceptions on possible contributions and qualities needed/required from ET in supporting and motivating seniors to perform physical activity | Explorative qualitative design using focus groups  Inductive qualitative analysis | 7 OA + HCPs  OA’s age: 66-82, both sexes, without cognitive impairments | CMT/UNMT: Digital technology-based motivation support |
| **25** | Elers Phoebe (2018) | New Zealand | To investigate how ET that connect OA to their informal and formal support networks could assist aging in place and enhance OA’s health and well-being | Qualitative approach using semi-structured interviews  Thematic analysis | 10 OA with at least one chronic health condition + 31 informal support network participants.  OA’s age: 75+, without cognitive impairments | UNMT: Home monitoring and Information and communication technologies (ICT) |
| **26** | Essén Anna (2008) | Sweden | To describe OA’s experience of electronic care surveillance in relation to their privacy | Qualitative approach using interviews  Thematic analysis | 17 OA  OA’s age: 68-96, both sexes, without cognitive impairments | UNMT: Monitoring service  It includes a monitoring device, worn on the wrist, that continuously collects “activity data” about the user. These data are transmitted to the care center and are accessed by the care personnel, in a graphical format as activity curves on a computer screen. The curves provide information about sleeping patterns, when the user leaves the house and takes off the device in order to detect changes in activity patterns, which can indicate emerging health problems. |
| **27** | Farivar Samira (2020) | Canada | To explore the role of OA’s cognitive age, subjective well-being, and perceptions regarding the complexity of ET, in terms of input data and output data, in their adoption behaviour | Mix-method approach using interviews [and survey]  Thematic analysis | 44 OA  OA’s age: 65+, both sexes, without cognitive impairments | UNMT: FitBit  It is a wearable device, attached to the user in order to monitor some aspects of their behaviours (e.g., physical activity, number of steps, distance, calories burned, and vital signs, such as heart rate and blood pressure) |
| **28** | Fisher Katharine (2019) | UK | To explore the value and adoption of digital technology by OA with sight loss | Mix-method approach using focus groups [and survey]  Framework analysis | 30 OA  OA’s age: 68-84, males with sight loss, but without cognitive impairments | CMT: Digital technologies  These include computers, smartphones, tablets |
| **29** | Göransson Carina (2018) | Sweden | To explore the experiences of using an app among OA with home-based health care and their nurses | Explorative qualitative approach using interviews  Thematic analysis | 17 OA  OA’s age: 70-101, both sexes, without cognitive impairments | CMT: Interaktor app  It is an interactive app for regular reporting of health concerns with direct and continuous access to self-care advice and graphs.  It includes a risk assessment model, so that alerts are sent to health care professionals for rapid management |
| **30** | Harrefors Christina (2010) | Sweden | To explore healthy older couples’ perceptions of using assistive technology services (ATSs) when in need of assistance with care | Qualitative approach using semi-structured interviews  Qualitative content analysis | 24 OA (12 couples)  OA’s age: 70+, both sexes, without cognitive impairments | CMT: ATSs  These includes tools that allow remote consultations, health examinations and other different technical devices when in need for help |
| **31** | Harris Maurita T. (2021) | USA | To explore the factors OA with hypertension initially consider when introduced to three new ET that might support their health self-management | Qualitative approach using semi-structured interviews  Qualitative data analysis | 23 OA  OA’s age: 65-84, both sexes, diagnosis of hypertension, without cognitive impairments | SAR: Multifunctional Healthcare Robot  It has two functions: to monitor blood pressure and to bring medication. The user can command the robot by talking to it. The robot has two arms, a touch screen monitor, and a removable pillbox tray. The SAR can check blood pressure by using a cuff and the touch screen monitor. |
| **32** | Hein Willius Andreas (2019) | Chile | To explore the concept of Personal, Portable, Electronic Health Device (DEPPAS) in general and the acceptability of the Personal Portable Device (PPD) that OA use | Exploratory qualitative approach using focus groups  Content analysis based on Technology Acceptance model | 20 OA  OA’s age: 65+, both sexes, without cognitive impairments | UNMT: DEPPAS  DEPPAS includes a PPD, a reader-writer device and a centralized data storage system.  With DEPPAS, healthcare professionals (i.e., disaster first responders) can access critical health information to make informed clinical decisions |
| **33** | Holender Anita (2018) | UK | To evaluate OA’s opinions on digital interventions to improve tablet taking delivered through smartphone, smartwatch, and ingestible sensor systems (ISSs) and to identify practical and perceptual barriers | Qualitative approach using focus groups  Thematic analysis | 12 OA  OA’s age: 65-75, with hypertensionwithout cognitive impairments | CMT: Mobile phones, apps  UNMT: smartwatches and ingestible sensor systems (ISSs)  These are a combination of wearable and ingestible sensors working in conjunction with mobile phones, computers and tablets to detect ingested medication |
| **34** | Holthe Torhild (2020) | Norway | To explore how OA in an assisted living facility experience the use of environmental sensors in their apartments | Qualitative approach using observations, focus groups and interviews  Inductive thematic analysis | 8 OA  OA’s age: 81-92, both sexes, without cognitive impairments | UNMT: Environmental sensors  The sensors are wirelessly connected to a computerized control box that transmitted signals to a secured server. The sensors include movement sensors, that registered when the resident entered or left different rooms, power effect sensors that registered power being used by the stove, coffee machine, TV or radio, and magnet sensors that registered whether doors/windows were open or closed. All sensors are connected to the push-button and loudspeaker, which issues an audio message if the button is activated. |
| **35** | Huang Tianyang (2020) | Taiwan | To explore the individual attributes of user factors on the user acceptance of companion robots and the influence of user factors on OA’s acceptance of companion robots.  To discuss the influences of educational level, professional background, occupation background, living status, gender, age and experience in using scientific and technological products, on the acceptance of companion robots | Mix method approach using semi-structured interviews [and questionnaire survey] | 6 OA  OA’s age: 66-75, both sexes, without cognitive impairments | SAR: Companion robots  These are machines that can understand and communicate in a manner similar to human communication (e.g., touch, hearing) |
| **36** | Hvalic-Touzery Simona (2021) | Slovenia | To explore reciprocity in informal carers’ and care recipients’ perceptions of telecare | Qualitative approach using semi-structured interviews  Thematic analysis | 11 OA + primary informal carers  OA’s age: 73-92, both sexes, without cognitive impairments | UNMT: Telecare equipment  The telecare equipment includes environmental sensors, like motion sensors and door sensors, smoke detectors and fall detectors, which function without manual actions. Sensor and fall detectors generate alerts when they detect data patterns which deviate from preestablished thresholds. There are also emergency pendants, that need the active role of the OA, who need to press the alarm in the case of an emergency. |
| **37** | Jo Tae Hee. (2021) | Korea | To investigate the OA’s perceptions of the Integrated Smart Home System (ISHS) | Qualitative approach using focus groups | 9 OA  OA’s age: 68-87, all females, without cognitive impairments | UNMT: ISHS  It includes blue low energy (BLE) smart bands, BLE receivers, and two types of environmental sensors.  ISHS provides the following functions: fall detection, healthcare monitoring, ADL (Activities of Daily Living) recognition, iAQ (indoor Air Quality) monitoring, and energy consumption monitoring |
| **38** | Johnson Austin (2021) | USA | To explore the barriers or facilitators to active surveillance of low-risk skin cancers using mHealth technology in OA | Qualitative approach using interviews  Thematic analysis | 33 OA + caregivers  OA’s Age: 65+, both sexes, without cognitive impairments | CMT: mHealth apps |
| **39** | Kärki Anne (2015) | Finland | To identify how independently living OA consider ICT/ICT AT as part of their life and how they consider that learning of ICT/ICT AT is taking place | Qualitative approach using interviews  Data based content analysis | 8 OA  OA’s age: 65-82, without cognitive impairments | CMT: ICT (information communication technologies) and ICT-AT  Examples are mobile phones, smartphone applications, computers |
| **40** | Knowles Bran (2018) | UK | To explore the stated attitudes of OA, exploring the implicit meanings conveyed by distrust, to better understand technology non-use among OA | Qualitative approach using focus groups  Grounded theory analysis | OA  OA’s age: 65+, both sexes, without cognitive impairments | CMT: Amazon, Google (search engine and maps), loyalty card, Uber and online banking  UNMT: activity trackers (e.g Fitbit), health trackers and home energy sensors (e.g Nest) |
| **41** | Kononova Anastasia (2019) | USA | To explore OA’s perceptions and uses of activity trackers at different points of use, in order to determine the factors to maintain tracker use and prevent users from discontinuing tracker usage | Qualitative approach using focus groups  Thematic analysis | 48 OA  OA’s age: 65-94, both sexes, with chronic conditions such arthritis, high blood pressure, heart disease, without cognitive impairments | UNMT: Wearable activity trackers  These tools include Garmin Vivofit2, Fitbit, Apple watch, Jawbone, Misfit Nike, Gear Fit.  Activity trackers are sensor-based wearable devices that automatically track and monitor various indicators of physical activity (e.g., steps taken, stairs climbed, duration and quality of sleep, pulse or heart rate, calories consumed or burned, and even mood) |
| **42** | Ladin Keren (2021) | USA | To identify patient, care partner, and nephrologists’ perceptions of the patient centeredness, benefits, disadvantages of telehealth compared to in-person visits | Qualitative approach using semi-structured interviews  Thematic analysis | 30 OA, with chronic conditions, including chronic kidney disease (CKD) + clinicians and care partners  OA’s age: 70+, both sexes, without cognitive impairments | CMT: Telehealth |
| **43** | Leikas Jaana (2018) | Finland | To understand the most pressing ethical concerns of OA and care professionals regarding ubiquitous home monitoring | Qualitative approach using focus groups | 8 OA + professionals in the home care field, project coordinators, teachers, students.  OA’s age: 70-85, both sexes, without cognitive impairments | UNMT: Monitoring technology  It includes sensors to create an intelligent ambient environment that gathers information on how well the inhabitant is performing her usual activities and reveals possible changes in daily habits |
| **44** | Lie Mabel L. S. (2016) | UK | To explore OA’s perspectives about the Shel home monitoring system | Qualitative approach using interviews  Thematic analysis | OA + monitors (relatives/friends)  OA’s age: 65+, both sexes with chronic age-related health conditions such as hypertension, hearing impairment, arthritis, and macular degeneration, but without cognitive impairments | UNMT: Shel home monitoring system  It is an example of ‘ambient assisted living’, which refers to environments sensitive to the presence of people. It provides automated home monitoring of OA’s activities in a non-invasive manner |
| **45** | McGlynn Sean A. (2014) | USA | To explore healthy OA’s acceptance of the PARO robot | Mix-method approach using interviews [and questionnaires] | 30 OA  OA’s age: 67-80, both sexes, without cognitive impairments | SAR: PARO  It is a baby harp seal mimic robot developed solely for therapeutic purposes. Its appearance, sounds and behaviours resemble that of a baby harp seal. Its function is only to elicit positive emotions like happiness and relaxation |
| **46** | As above (2017) | USA | To investigate attitudes about, engagement with, and emotions toward PARO for independently living OA | Mix-method approach using semi-structured interviews [and questionnaires]  Thematic analysis | 30 OA  OA’s age: 67-80, both sexes, without cognitive impairments | SAR: PARO  It is a seal-robot, specifically designed for therapy, to promote engagement. It is designed for long-term usage and its function is only to elicit positive emotions like happiness and relaxation |
| **47** | Neves Barbara B. (2021) | Australia | To understand, from a sociological point of view, how frail OA living in care settings learn to use ET in later life | Qualitative approach using semi-structured interviews  Thematic analysis- | 12 OA + study partner (relative or friend)  OA’s Age: 74-95, both sexes, without cognitive impairments | CMT: a communication app  The app allows you to send audio, images and videos as well as text messages to a list of contacts that appears as a digital photo-album. |
| **48** | As above (2021) | Australia | To explore instances when technology-based interventions were limiting or failed, seeming to enhance rather than lessen loneliness | A mix-method approach using observations and semi-structured interviews [and scales and tests].  Thematic analysis | OA  OA’s age: 65+, both sexes, without cognitive impairments | CMT: Tablet based communication App  The app allows for asynchronous multimedia communication, sending and receiving text, video, audio, and picture messages. |
| **49** | Niemela Marketta. (2021) | Finland | To study how using a telepresence robot influences the resident, family members and care workers at a facility.  To understand what challenges and solutions there are for wider adoption of such robots in residential care | Empirical study based on qualitative approach using observations and interviews | 3 OA + family members and care workers  OA’s age: 83-93, both sexes, without clinically relevant cognitive impairments | SAR: Double  It is a telepresence robot (or virtual presence or remote presence robot), a remote-controlled robotic device which enables a person not just to be virtually present, interact and socially participate from a remote location, but also physically move in the robot’s local environment, so allowing the remote user to take more control over her/his presence.  By pressing a separate big button the resident can send an SMS request to the mobile phone of the family member to ask her/him to open the telepresence connection. The secondary users used laptops to open the video connection to the robot |
| **50** | Ostrowski Anastasia K. (2021) | USA | To gather design criteria for social robots | Qualitative approach using interviews/co-design process  Ground theory approach | 28 OA  OA’s Age: 70-94, both sexes, without cognitive impairments | UNMT: Amazon Echo, Google Home, Apple Siri, Microsoft Cortana  These are voice agent technologies, that uses speech as a main modality for interaction  SAR: Jibo  It is a social robot, that moves, rotates and has a touchscreen interface |
| **51** | Park Yeon-Hwan (2019) | Korea | To identify OA’s needs and acceptance of robot services that have the potential to address their self-perceived needs and deficiencies | Mix-method approach using focus groups [and surveys]  Thematic analysis | 23 OA  OA’s age: 66-85, both sexes, without cognitive impairments | SAR: Robot service |
| **52** | Peek Sebastiaan T. M. (2016) | The Netherlands | To explore which factors influence the level of use of various types of technology by OA who are aging in place | Qualitative explorative approach using semi-structured interviews  Thematic analysis | 53 OA  OA’s age: 68-95, both sexes, without cognitive impairments | CMT: ICT devices, telephones  UNMT: Smart Home/e-Health  Smart Home technologies include emergency help systems, vital sign monitoring and fall detection systems  e-Health encompasses a broad range of technologies, e.g., online tools to support self-management of chronic conditions |
| **53** | As above (2019) | The Netherlands | To explore changes and stability in the use of technologies by independent-living OA, by using a dynamical systems theory approach | Perspective longitudinal qualitative field study using semi-structured interviews  Thematic analysis | 33 OA  OA’s age: 70 -80, both sexes, without cognitive impairments | CMT: Technology, ICT devices |
| **54** | Pigini Lucia (2012) | Italy | To explore the needs and perceptions of OA, family members and professionals in order to generate specific user requirements and concrete scenarios for the development of a new technological assistive device fulfilling as much as possible the real needs of the potential users | Mix-method approach using focus groups [and questionnaires] | 22 OA + relatives, professional caregivers and health professionals  OA’s age: 65-90, both sexes, without cognitive impairments | SAR: Semi-autonomous robot |
| **55** | Pol Margriet (2016) | The Netherlands | To explore the perspectives of OA regarding the use of sensor monitoring technologies in their daily lives | Qualitative interpretative phenomenological approach using semi-structured interviews  Interpretative phenomenological analysis | 23 OA  OA’s age: 68-94, both sexes, without cognitive impairments | UNMT: Sensor monitoring system  It includes passive infrared motion sensors (to detect motion), magnetic contact sensors on doors and cabinets (to measure whether doors are opened or closed) and a flush sensor in the toilet (to measure the toilet being flushed). The sensor data are analyzed by an intelligent software program using machine learning techniques, that searches for activities of daily functioning and patterns of daily functioning (e.g., toileting, bathing or bed rest, but also more complex activities such as preparing kitchen activities). |
| **56** | Portet François (2013) | France | To explore the acceptance and fear of home automation based on voice command | Qualitative approach using Interviews | 8 OA + relatives and caregivers  Mean age of OA: 79, both sexes, without cognitive impairments | UNMT: The DOMUS Smart Home  It is a thirty square meters suite flat, with sensors and effectors, dedicated to the observation and the measurement of users’ interactions with the ambient intelligence of the environment. |
| **57** | Pripfl Jürgen (2016) | Austria | To raise the Social Service Robots (SSRs) to a level that allows the robot to be fully autonomously deployed in the private homes of OA  To evaluate technology market readiness, utility, usability and affordability under real-world conditions | Mix method approach using interviews [and questionnaires]  Qualitative data analysis | 7 OA  OA’s age:75-88, both sexes, without cognitive impairments | SAR: HOBBIT Robot  It is a platform with a floor-parallel depth camera, a head screen with an RGB-D (Red Green Blu-Depth) camera, a touch screen in front of the torso and an arm with a gripper.  Hobbit interacts with the user via a multimodal user interface (MMUI) which consists of a graphical user interface (GUI) with touch input, automatic speech recognition (ASR), text to speech (TTS) and gesture recognition interface (GRI). The SAR provides entertainment (e.g., radio, music, audio books, games, pre-installed web services and fitness function), reminders, videophone service, access to an ambient assisted living (AAL) environment (e.g. call buttons) and emergency call features. Its functions include automatic fall detection, handling emergencies (e.g., calming dialogues and communication with relatives) and supportive fall prevention measures (e.g. transporting small items, picking up objects from the floor, searching for objects as programmed by the user) |
| **58** | Sanchez Veralia Gabriela (2019) | Norway | To explore attitudes and perspectives about welfare technology among OA living alone | Explorative qualitative approach using semi-structured interviews  Content analysis | 9 OA  OA’s age: 75-91, both sexes, without cognitive impairments | UNMT: Welfare technology  It is technology used for environmental control, safety and wellbeing (often referred to as 'ambient assisted living' outside of Scandinavia). It can contribute to facilitating sustainable healthcare |
| **59** | Stack Emma (2016) | UK | To observe OA in their own homes to identify what types of sensors, and in which locations, were capable of monitoring mobility and balance in a way that would be acceptable to participants and meet the researchers’ needs | Qualitative approach using observations | 5 OA  OA’s age: 71-79, both sexes, with Parkinson's diagnosis, without cognitive impairments | UNMT: in-home sensors  The sensors include video, Kinect camera and wearable devices, that record movement patterns (e.g., habitual activities), behaviours (likely to increase or decrease fall risk), locations and actions associated with (historic or observed) falls and near-misses and fear of falling |
| **60** | Steele Robert (2009) | Australia | To provide information on the perceptions of OA towards current wireless sensor network (WSN) designs as well as to facilitate a communication channel between users and researchers, informing the research community on applications and functionalities that users deem as either desirable, inadequate or in need of further development | Explorative qualitative design using focus groups  Thematic analysis | 13 OA  OA’s age: 65+, both sexes, without cognitive impairments | UNMT: WSN-based systems  These systems include a series of sensor nodes, AKA motes. Each mote can facilitate the collection of environmental and structural sensory information (e.g., weight, blood sugar, blood oxygen level, ECG information, EEG information, sound, temperature, humidity, light-intensity, vibration, and acceleration) |
| **61** | Thilo Friederike J.S. (2017) | Switzerland | To involve community-dwelling OA in device design of a fall detection sensor, including its smartphone application, through the evaluation of the mock-up | Qualitative descriptive study using focus groups  Deductive approach analysis | 22 OA  OA’s age: 75-89, both sexes, without cognitive impairments | CMT: Smartphone  UNMT: Fall detection sensor  It is a waterproof device, wearable >24 hours and is fixed by a patch on the torso. It is connected via Bluetooth to a smartphone application.  When a fall has occurred, the application will open automatically. An alert may be stopped according to an individually adjustable timeframe, illustrated to a closing cycle. As soon as the alert is sent to the contact persons, the application window changes and indicates that help is coming. An alert is emitted, indicating the location (GPS – Global Positioning System) of the fallen person, which is automatically transmitted via SMS successively to predefined contact persons until one person answers the alert |
| **62** | Thomas Lisa (2013) | UK | To understand attitudes to Location Based Services (LBS) technologies in OA | Mix method approach using in depth interviews [and questionnaires]  Thematic analysis | 20 OA  OA’s age: +65, both sexes, without cognitive impairments | CMT: Location based services (LBS) technology  Examples are applications such as Yelp, Twitter, AroundMe, which share location information and can be assessed with a mobile phone |
| **63** | Tsai Hsin-Yi Sandy (2015) | USA | To examine OA’s technology adoption to narrow the gap of technology use (AKA digital divide) between OA and younger generations | Qualitative approach using semi-structured interviews | 21 OA  OA’s age: 69-91, both sexes, without cognitive impairments | CMT: Tablets  Examples are iPad and Kindle Fire |
| **64** | Van Houwelingen Cornelis T. M. (2018) | The Netherlands | To understand community-dwelling OA’s readiness to receive telehealth by studying their intention to use videoconferencing and capacities or incapacities to use digital technology in daily life as indicators | Mix-method approach using observations [and cross-sectional survey]  Interpretative phenomenological analysis | 15 OA  OA’s age: 65-87, both sexes, without cognitive impairments | CMT: Telehealth  It indicates healthcare delivery remotely through the use of digital technology such as videoconferencing |
| **65** | Vandemeulebroucke Tijs (2020) | Belgium | To explore what community-dwelling OA perceive as ethical issues in using SAR in aged-care settings | Qualitative approach using focus groups  Grounded theory | 59 OA  OA’s age: 70+, both sexes, without cognitive impairments | SAR: Alice  It has a small humanoid body and a humanlike face, mimicking a young girl. Although it cannot move its body, the robot can interact with its environment by talking |
| **66** | Vaportzis Eleftheria (2017) | UK | To understand the perceptions of, and barriers to, interacting with tablets in healthy OA who were novice tablet users | Qualitative approach using focus group  Inductive thematic analysis | 18 OA  OA’s age: 65-76, both sexes, cognitively intact | CMT: tablets  The following five touchscreen tablets were chosen: Asus TF103CX, Asus Google Nexus 7, Samsung Galaxy Tab 3 (8’’), Apple iPad Mini, Samsung Galaxy Tab 3 (10.1’’). |
| **67** | Walsh Kieran (2011) | Ireland | To explore current and prospective users’ perceptions of and preferences for the use of ICT technology in community social care settings | Qualitative approach using focus groups | 15 OA + general population (18-84 years old)  OA’s age: 65+, both sexes, without cognitive impairments | CMT: Telehealth  It refers to the application of medical practice by telematic means  UNMT: Telecare and Smart Home  Telecare involves community social alarm services; sensory technology; home health monitoring and care provider and rehabilitation technology. Smart housing describes the electronic and computer-controlled integration of many activities and devices within the home |
| **68** | Wang Shengzhi (2019) | USA | To involve residents of a local continuing care senior housing community (CCSHC) in conversations about ET that might facilitate their continued independent living status; to assess their privacy attitudes and preferences; and to identify whether OA would be interested in co-designing technologies moving forward and if so, how to foster next steps | Mix-method approach using focus groups [and survey]  Grounded theory approach | 31 OA  OA’s age: 67-94, both sexes, without cognitive impairments | CMT/UNMT: different kind of technologies based on AI |
| **69** | Wild Katherine (2008) | USA | To explore themes and to assess positive and negative responses to unobtrusive in-home monitoring from the perspectives of both the OA and their family members or friends, as potential users | Qualitative approach using focus groups  Thematic analysis | 31 OA + family members/friends  OA’s age: 66-91, both sexes, without cognitive impairments | UNMT: Health monitoring system/In-home system  It is a system based on movement detection by infrared sensors. By identifying “usual” and “unusual” patterns of activity over time, it is possible to make inferences about changes in health conditions |
| **70** | Wilson-Nash Carolyn (2022) | UK | To explore the lived experiences of OA who have newly acquired a portable digital technology, recording their interactions with these devices across an extended period | Qualitative approach using self-report diary  Thematic analysis | 12 OA  OA’s age: 65+, both sexes, without cognitive impairments | CMT: Portable digital technologies Examples are tablets, smartphones, laptops and e-readers |

^a^ AI: Artificial Intelligence; AKA: Also Known As; AT: Assistive Technologies; CMT: Conventional Monitoring Techniques; ECG: Electrocardiogram; EEG: Electroencephalogram; ET: Emerging Technologies; ICT: Information and Communication Technologies; MMS: Multimedia Messaging Service; OA: Older Adults; SAR: Socially Assistive Robots; SMS: Short Message Service; UNMT: Unconventional Monitoring Techniques.

**Reference List**

[1] Åkerlind C, Martin L, Gustafsson C. eHomecare and safety: The experiences of older patients and their relatives. Geriatric Nursing. 2018 Mar 1;39(2):178-85.

[2] Bajones M, Fischinger D, Weiss A, De La Puente P, Wolf D, Vincze M, Körtner T, Weninger M, Papoutsakis K, Michel D, Qammaz A, Panteleris P, Foukarakis M, Adami I, Ioannidi D, Leonidis A, Antona M, Argyros A, Mayer P, Panek P, Eftring H, Frennert S. Results of Field Trials with a Mobile Service Robot for Older Adults in 16 Private Households. Acm Transactions on Human-Robot Interaction. 2020;9(2):1-27.

[3] Baric V, Andreassen M, Öhman A, Hemmingsson H. Using an interactive digital calendar with mobile phone reminders by senior people - a focus group study. BMC Geriatrics. 2019; 19(116):1-11.

[4] Bevilacqua R, Felici E, Cavallo F, Amabili G, Maranesi E. Designing Acceptable Robots for Assisting Older Adults: A Pilot Study on the Willingness to Interact. Int J Environ Res Public Health. 2021;18(10686):1-9.

[5] Bian C, Bing Y, Hoonakker A, Mihailidis A. Attitudes and perspectives of older adults on technologies for assessing frailty in home settings: a focus group study. BMC Geriatrics. 2021;21(298): 1-13.

[6] Boström M, Kjellström S, Björklund A. Older persons have ambivalent feelings about the use of monitoring technologies. Technology & Disability. 2013;25(2): 117-125.

[7] Broadbent E, Tamagawa R, Patience A, Knock B. Attitudes towards health-care robots in a retirement village. Australasian Journal on Ageing. 2012;31(2): 115-120.

[8] Cabrita M, Tabak M, Vollenbroek-Hutten M. Older Adults' Attitudes Toward Ambulatory Technology to Support Monitoring and Coaching of Healthy Behaviors: Qualitative Study. JMIR Aging. 2019;2(1):1-12.

[9] Cajita MI, Hodgson NA, Lam KW, Yoo S, Han HR. Facilitators and Barriers to mHealth Adoption in Older Adults with Heart Failure. CIN: Computer, Informatics, Nursing. 2018;36(8):376-382.

[10] Chang CP, Lee TT, Mills ME.  Experience of Home Telehealth Technology in Older Patients With Diabetes. CIN: Computers, informatics, Nursing. 2017;35(10):530-537.

[11] Choi YK, Thompson HJ, Demiris G. Internet-of-Things Smart Home Technology to Support Aging-in-Place: Older Adults' Perceptions and Attitudes. Journal of Gerontological Nursing. 2021;47(4):15-21.

[12] Chung J, Thompson HJ, Joe J, Hall A, Demiris G. Examining Korean and Korean American older adults' perceived acceptability of home-based monitoring technologies in the context of culture. Informatics for Health & Social Care. 2017;42(1):61-76.

[13] Coghlan S, Waycott J, Lazar A, Barbosa Neves B. Dignity, Autonomy, and Style of Company: Dimensions Older Adults Consider for Robot Companions. Proc ACM Hum Comput Interact. 2021;5(CSCW1):1-24.

[14] Courtney KL. Privacy and senior willingness to adopt smart home information technology in residential care facilities. Methods of Information in Medicine. 2008;47(1):76-81.

[15] Courtney KL, Demeris G, Rantz M, Skubic M. Needing smart home technologies: the perspectives of older adults in continuing care retirement communities. Informatics in Primary Care. 2008;16:195-201.

[16] Daniele K, Marcucci M, Cattaneo C, Borghese NA, Zannini L. How Prefrail Older People Living Alone Perceive Information and Communications Technology and What They Would Ask a Robot for: Qualitative Study. Journal of Medical Internet Research. 2019;21(8):1-12.

[17] Davenport RD, Elzabadani H, Johnson JL, Helal AS, Mann WC. Pilot live-in trial at the GatorTech Smarthouse. Topics in Geriatric Rehabilitation. 2007;23(1):73-84.

[18] Demiris G, Rantz MJ, Aud MA, Marek KD, Tyrer HW, Skubic M, Hussam AA. Older adults' attitudes towards and perceptions of 'smart home' technologies: a pilot study,. Medical Informatics & the Internet in Medicine. 2004;29(2):87-94.

[19] Demiris G, Oliver DP, Dickey G, Skubic M, Rantz M. Findings from a participatory evaluation of a smart home application for older adults. Technol Health Care. 2008;16(2):111-118.

[20] Demiris G, Hensel BK, Skubic M, Rantz M. Senior residents' perceived need of and preferences for 'smart home' sensor technologies. International Journal of Technology Assessment in Health Care. 2008;24(1):120-124.

[21] Dermody G, Fritz R, Glass C, Dunham M, Whitehead L. Factors influencing community‐dwelling older adults’ readiness to adopt smart home technology: A qualitative exploratory study. Journal of Advanced Nursing. 2021 Dec;77(12):4847-61.

[22] Deutsch I, Erel H, Paz M, Hoffman G, Zuckerman O. Home robotic devices for older adults: Opportunities and concerns. Computers in Human Behavior. 2019;98:122-133.

[23] Ehn M, Eriksson LC, Åkerberg N, Johansson AC. Activity Monitors as Support for Older Persons' Physical Activity in Daily Life: Qualitative Study of the Users' Experiences. JMIR Mhealth and Uhealth. 2018;6(2):1-15.

[24] Ehn M, Johansson AC, Revenäs Å. Technology-Based Motivation Support for Seniors' Physical Activity-A Qualitative Study on Seniors' and Health Care Professionals' Views. International Journal of Environmental Research and Public Health. 2019;16(13):1-20.

[25] Elers P, Hunter I, Whiddett D, Lockhart C, Guesgen H, Singh A. User Requirements for Technology to Assist Aging in Place: Qualitative Study of Older People and Their Informal Support Networks. JMIR Mhealth and Uhealth. 2018;6(6):1-7.

[26] Essen A. The two facets of electronic care surveillance: an exploration of the views of older people who live with monitoring devices. Social Science&Medicine. 2008;67(1):128-136.

[27] Farivar S, Abouzahra M, Ghasemaghaei M. Wearable device adoption among older adults: A mixed-methods study. International Journal of Information Management. 2020;55:1-14.

[28] Fisher K, Easton K. The meaning and value of digital technology adoption for older adults with sight loss: A mixed methods study. Technology & Disability. 2019;30(4):177-184.

[29] Göransson C, Eriksson I, Ziegert K, Wengström Y, Langius‐Eklöf A, Brovall M, Kihlgren A, Blomberg K. Testing an app for reporting health concerns-Experiences from older people and home care nurses. International Journal of Older People Nursing. 2018;13(2):e12181.

[30] Harrefors C, Axelsson K, Sävenstedt S. Using assistive technology services at differing levels of care: healthy older couples' perceptions. Journal of Advanced Nursing. 2010;66(7):1523-1532.

[31] Harris MT, Rogers WA. Developing a Healthcare Technology Acceptance Model (H-TAM) for Older Adults with Hypertension. Ageing & Society. 2021;1-21.

[32] Hein Willius A, Torres Hidalgo M, Arroyo Zuñiga P, Quezada Venegas M, Arriagada Díaz C, Valenzuela Abarca E, San Martín Gutierrez E, Bedregal P. An Acceptability Study of A Personal Portable Device Storing Critical Health Information To Ensure Treatment Continuity Of Home-Dwelling Older Adults In Case Of A Disaster." Patient Prefer Adherence. 2019;13:1941-1949.

[33] Holender A, Sutton S, De Simoni A. Opinions on the use of technology to improve tablet taking in >65-year-old patients on cardiovascular medications. J Int Med Res. 2018;46(7):2754-2768.

[34] Holthe T, Halvorsrud L, Lund A. A critical occupational perspective on user engagement of older adults in an assisted living facility in technology research over three years. Journal of Occupational Science. 2020;27(3):376-389.

[35] Huang TY, Huang C. Elderly's acceptance of companion robots from the perspective of user factors. Universal Access in the Information Society. 2020;19:935-948.

[36] Hvalic-Touzery S, Smole-Orehek K, Dolnicar V. Exploring reciprocity in perceptions on telecare within the informal carer-care receiver dyad. Teorija in Praksa. 2021;58(3):840-859.

[37] Jo TH, Ma JH, Cha SH. Elderly Perception on the Internet of Things-Based Integrated Smart-Home System. Sensors. 2021;21(1284):1-28.

[38] Johnson A, Shukla N, Halley M, Nava V, Budaraju J, Zhang L, Linos E. Barriers and facilitators to mobile health and active surveillance use among older adults with skin disease. Health Expectations. 2021;24:1582-1592.

[39] Kärki A, Sallinen M, Kuusinen J. How to live independently with or without technology? Stud Health Technol Inform. 2015;217:306-310.

[40] Knowles B, Hanson VL. Older Adults' Deployment of 'Distrust'. ACM Transactions on Computer-Human Interaction. 2018;25(4):1-25.

[41] Kononova A, Li L, Kamp K, Bowen M, Rikard RV, Cotton S, Peng W. The Use of Wearable Activity Trackers Among Older Adults: Focus Group Study of Tracker Perceptions, Motivators, and Barriers in the Maintenance Stage of Behavior Change. JMIR Mhealth Uhealth. 2019;7(4):1-16.

[42] Ladin K, Porteny T, Perugini JM, Gonzales KM, Aufort KE, Levine SK, Wong JB, Isakova T, Rifkin D, Gordon EJ, Rossi A, Koch-Weser S, Weiner DE. Perceptions of Telehealth vs In-Person Visits Among Older Adults With Advanced Kidney Disease, Care Partners, and Clinicians. JAMA Network Open. 2021;4(12):1-12.

[43] Leikas J, Kulju M. Ethical consideration of home monitoring technology: A qualitative focus group study. Gerontechnology. 2018;17(1):38-47.

[44] Lie MLS, Lindsay S, Brittain K. Technology and trust: older people's perspectives of a home monitoring system. Ageing & Society. 2016;75(3):1-25.

[45] McGlynn SA, Kemple SC, Mitzner TL, King CH, Rogers WA. Understanding Older Adults' Perceptions of Usefulness for the Paro Robot. Proc Hum Factors Ergon Soc Annu Meet. 2014;58(1):1914-1918.

[46] McGlynn SA, Kemple S, Mitzner TL, King CHA, Rogers WA. Understanding the potential of PARO for healthy older adults. International Journal of Human-Computer Studies. 2017;100:33-47.

[47] Neves BB, Mead G. Digital Technology and Older People: Towards a Sociological Approach to Technology Adoption in Later Life. Sociology-the Journal of the British Sociological Association. 2021;55(5):888-905.

[48] Neves BB, Waycott J, Maddox A. When Technologies are Not Enough: The Challenges of Digital Interventions to Address Loneliness in Later Life. Sociological Research Online. 2023;28(1):150-170.

[49] Niemela M, van Aerschot L, Tammela A, Aaltonen, Lammi H. Towards Ethical Guidelines of Using Telepresence Robots in Residential Care. International Journal of Social Robotics. 2021;13(3):431-439.

[50] Ostrowski AK, Harrington CN, Breazeal C, Won Park H. Personal Narratives in Technology Design: The Value of Sharing Older Adults' Stories in the Design of Social Robots. Frontiers in Robotics and AI. 2021;8:1-17.

[51] Park YH, Chang HK, Lee MH, Lee SH. Community-dwelling older adults' needs and acceptance regarding the use of robot technology to assist with daily living performance. BMC Geriatrics. 2019;19(208):1-9.

[52] Peek STM, Luijkx KG, Rijnaard MD, Nieboer ME, van der Voort CS, Aarts S, van Hoof J, Vrijhoef HJM, Wouters EJM. Older Adults' Reasons for Using Technology while Aging in Place. Gerontology. 2016;62(2):226-237.

[53] Peek STM, Luijkx KG, Vrijhoef HJM, Nieboer ME, Aarts S, van der Voort CS, Rijnaard MD, Wouters EJM. Understanding changes and stability in the long-term use of technologies by seniors who are aging in place: a dynamical framework. BMC Geriatrics. 2019;19(236):1-13.

[54] Pigini L, Facal D, Blasi L, Andrich R. Service robots in elderly care at home: Users' needs and perceptions as a basis for concept development. Technology & Disability. 2012;24(4):303-311.

[55] Pol M, van Nes F, van Hartingsveldt M, Buurman B, de Rooij S, Kröse B. Older People's Perspectives Regarding the Use of Sensor Monitoring in Their Home. The Gerontologist. 2016;56(3):485-493.

[56] Portet F, Vacher M, Golanski C, Roux C, Meillon B. Design and evaluation of a smart home voice interface for the elderly: acceptability and objection aspects. Personal and Ubiquitous Computing. 2013;17:127-144.

[57] Pripfl J, Körtner T, Batko-Klein D, Hebesberger D, Weninger M, Gisinger C. Social service robots to support independent living Experiences from a field trial. Zeitschrift Fur Gerontologie Und Geriatrie. 2016;49:282-287.

[58] Sanchez VG, Anker-Hansen C, Taylor I, Eilertsen G. Older People's Attitudes and Perspectives Of Welfare Technology In Norway. Journal of Multidisciplinary Healthcare. 2019;12:841-853.

[59] Stack E, King R, Janko B, Burnett M, Hammersley N, Agarwal V, Hannuna S, Burrows A, Ashburn A. Could In‐Home Sensors Surpass Human Observation of People with Parkinson’s at High Risk of Falling? An Ethnographic Study. BioMed research international. 2016;2016:1-10.

[60] Steele R, Lo A, Secombe C, Wong YK. Elderly persons’ perception and acceptance of using wireless sensor networks to assist healthcare. International journal of medical informatics. 2009 Dec 1;78(12):788-801.

[61] Thilo FJ, Bilger S, Halfens RJG, Schols JMGA, Hahn S. Involvement of the end user: exploration of older people's needs and preferences for a wearable fall detection device - a qualitative descriptive study. Patient Prefer Adherence. 2017;11:11-22.

[62] Thomas L, Little L, Briggs P, McInnes L, Jones E, Nicholson J. Location tracking: views from the older adult population. Age and ageing. 2013 Nov 1;42(6):758-63.

[63] Tsai HS, Shillair R, Cotton SR, Winstead V, Yost E. Getting Grandma Online: Are Tablets the Answer for Increasing Digital Inclusion for Older Adults in the US? Educational Gerontology. 2015;41(10): 695-709.

[64] Van Houwelingen CTM, Ettema RGA, Antonietti MGEF, Kort HSM. Understanding Older People's Readiness for Receiving Telehealth: Mixed-Method Study. Journal of Medical Internet Research. 2018;20(4): 1-17.

[65] Vandemeulebroucke T, Dierckx de Casterlé B, Welbergen L, Massart M, Gastmans C. The ethics of socially assistive robots in aged care. A focus group study with older adults in Flanders, Belgium. The Journals of Gerontology: Series B. 2020 Nov;75(9):1996-2007.

[66] Vaportzis E, Clausen MG, Gow AJ. Older Adults Perceptions of Technology and Barriers to Interacting with Tablet Computers: A Focus Group Study. Frontiers in Psychology. 2017;8(1687):1-11.

[67] Walsh K, Callan A. Perceptions, Preferences, and Acceptance of Information and Communication Technologies in Older-Adult Community Care Settings in Ireland: A Case-Study and Ranked-Care Program Analysis. Ageing International. 2011;36: 102-122.

[68] Wang SZ, Bolling K, Mao W, Reichstadt J, Jeste D, Kim HC, Nebeker C. Technology to Support Aging in Place: Older Adults' Perspectives. Healthcare. 2019;7(60):1-18.

[69] Wild K, Boise L, Lundell J, Foucek A. Unobtrusive in-home monitoring of cognitive and physical health: reactions and perceptions of older adults. Journal of Applied Gerontology. 2008;27(2):181-200.

[70] Wilson-Nash C, Tinson J. 'I am the master of my fate': digital technology paradoxes and the coping strategies of older consumers. Journal of Marketing Management. 2022;38(3-4):248-278.
