# Supplementary material for: Emerging Technologies and Vulnerabilities in Older Adults Without Cognitive Impairments: Systematic Review of Qualitative Evidence
Source: Interact J Med Res. 2026 Feb 19;15:e69676. doi: 10.2196/69676 (PMC12919910; doi:10.2196/69676)
Supplement: Multimedia Appendix 6 [file ijmr-v15-e69676-s006.docx]

**Multimedia Appendix 6.** Example of the Qualitative Analysis Guide of Leuven conceptual scheme.

**Reference: Ostrowski AK, Harrington CN, Breazeal C, Won Park H. Personal Narratives in Technology Design: The Value of Sharing Older Adults' Stories in the Design of Social Robots. Frontiers in Robotics and AI. 2021;8:1-17.**

| **Focus** | “Analysis of a year-long co-design project with 28 older adults focusing on storytelling as a means of gathering design criteria for social robots.” (p.2) | | |
| --- | --- | --- | --- |
| **Aim** | See above | | |
| **Technology** | UNMT/SAR | Five popular voice agent technologies, including a social robot: 1) Amazon Echo, 2) Google Home, 3) Apple Siri, 4) Microsoft Cortana, and 5) Jibo | “Jibo is a social robot that moves, rotates, and has a touchscreen interface.” (p.4)  “Voice agent” was used as an overarching term for any intelligent technology that uses speech as a main modality for interaction.” (p.5) |
| **Population** | Older adults  Age: 70-94, both sexes | | Using technologies |
| **Type of Research** | Qualitative: interviews, storytelling across the co-design process | | |
| **Place** | U.S. | | |
| **Time** | April 2019-april 2020 | | |
| **Linked to COVID-19 Pandemic** | NO | | |
| **Theoretical Approaches//Moral Theories** | NO | |  |
| **Reference to vulnerability and akin concepts** | Implicit | | |
| **Results in relation to dimensions of aged care vulnerability (Sanchini et al. BMC 2022)** | *Dimensions of aged care vulnerability*  *Physical vulnerability (PHV)*  A robot tame PHV, meant as non-pathological physical/physiological bodily deterioration related to ageing because of **its monitoring function** and because **it makes** **feel older adults secure at home.**  “Safety was largely described as **physical safety in terms of home security or fall detection**. P12 told a story of how a robot could **help them feel secure in their home**: “I’m asleep, there’s a noise outside, but it doesn’t wake me up, but it wakes up the robot. The robot says, ‘(P12), wake up, there’s a noise outside.’ It seems like a useful idea that if I’m sleeping, **and I could have a robot that detects something unusual that I would like to be alerted to** … I hear a fire truck coming, I hear a siren, I hear a buzzer, I hear the windows rattling. You could program the thing, and say **if I have a sound like somebody trying to get through the window, please wake me up** … I’m trying to say, I think there are people around here who are probably very worried about their personal security.” - P12 (initial interview) A robot programmed to alert an emergency service of a security breech **could help older adults feel more secure in their home and also know that there was something in place to monitor their safety**” (p.7).  *Relational/Interpersonal Vulnerability (RV)*  *Relationships between OAs, caregivers and family members*  A robot exacerbates RV, in the sense of interdependence in real-world settings, because some participants were **afraid it could replace human presence.**  “(…) **If it is a robot … It’s so inhumane. I’d prefer to say some words to her (family member), that are humane, like good morning. I wouldn’t use the robot in the first place. I would use some way that in this family, we communicate with one another, when I’m in my room”** P22 (p.8).  “(…) **Older adults’ concerns around technology that may draw people away from building in person physical relationships and push them to interact with others superficially in the digital world.** While a robot has a physical form, participants emphasized that they **would not want the robot to be all-consuming and, instead, promote human-human interactions**” (p.8).  *Human-robot interaction (HRI)*  A robot has a negative impact on HRI, because “(…) **If it is a robot … It’s so inhumane. I’d prefer to say some words to her (family member), that are humane, like good morning. I wouldn’t use the robot in the first place. I would use some way that in this family, we communicate with one another, when I’m in my room”** P22 (p.8).  *Moral vulnerability (MV)*  *Privacy/control and surveillance*  A robot tames MV, because participants felt monitored in the positive way, “I’m asleep, there’s a noise outside, but it doesn’t wake me up, but it wakes up the robot. The robot says, ‘(P12), wake up, there’s a noise outside.’… It seems like a useful idea that if I’m sleeping, **and I could have a robot that detects something unusual that I would like to be alerted to** … I hear a fire truck coming, I hear a siren, I hear a buzzer, I hear the windows rattling. You could program the thing, and say **if I have a sound like somebody trying to get through the window, please wake me up** … I’m trying to say, I think there are people around here who are probably very worried about their personal security.” - P12 (initial interview) A robot programmed to alert an emergency service of a security breech **could help older adults feel more secure in their home and also know that there was something in place to monitor their safety**” (p.7). | | |
| **Means to address vulnerabilities (as suggested by older adults)** | N.A. | | |
