# Supplementary material for: Emerging Technologies and Vulnerabilities in Older Adults Without Cognitive Impairments: Systematic Review of Qualitative Evidence
Source: Interact J Med Res. 2026 Feb 19;15:e69676. doi: 10.2196/69676 (PMC12919910; doi:10.2196/69676)
Supplement: Multimedia Appendix 8 [file ijmr-v15-e69676-s008.docx]

**Multimedia Appendix 8.** Quality assessment of included articles (CASP^a^).

|  | Author | (1) Was there a clear statement of the aims of the research? | (2) Was qualitative methodology appropriate? | (3) Was the research design appropriate to address the aim? | (4) Was the recruitment strategy appropriate to the aims? | (5) Was the data collected in a way that addressed the research issue? | (6) Was the relationship between the researcher and participants adequately considered? | (7) Were ethical issues taken into consideration? | (8) Was the data analysis sufficiently rigorous? | (9) Was there a clear statement of finding? | (10) How valuable was the research? | Overall |
| --- | --- | --- | --- | --- | --- | --- | --- | --- | --- | --- | --- | --- |
| Included qualitative publications | | | | | | | | | | | | |
| 1. | [1] | Y^b^ | Y | N^c^ | Y | Y | N | Y | Y | Y | Y | High |
| 2. | [3] | Y | Y | N | Y | Y | N | Y | Y | Y | Y | High |
| 3. | [5] | Y | Y | N | Y | Y | N | Y | Y | Y | Y | High |
| 4. | [6] | Y | Y | Y | Y | Y | N | Y | Y | Y | Y | High |
| 5. | [8] | Y | Y | Y | Y | Y | U^d^ | Y | Y | Y | Y | High |
| 6. | [9] | Y | Y | N | U | Y | N | Y | Y | Y | Y | Moderate |
| 7. | [10] | Y | Y | N | Y | Y | N | Y | Y | Y | Y | High |
| 8. | [11] | Y | Y | N | Y | Y | N | Y | Y | Y | Y | High |
| 9. | [12] | Y | Y | N | Y | Y | N | Y | Y | Y | Y | High |
| 10. | [13] | Y | Y | N | Y | Y | N | Y | Y | Y | Y | High |
| 11. | [14] | Y | Y | Y | Y | Y | N | U | U | Y | Y | Moderate |
| 12. | [15] | Y | Y | Y | Y | Y | N | Y | Y | Y | Y | High |
| 13. | [16] | Y | Y | Y | Y | Y | Y | Y | U | Y | Y | High |
| 14. | [18] | Y | Y | N | Y | Y | N | Y | U | Y | Y | Moderate |
| 15. | [19] | Y | Y | U | U | Y | N | Y | N | Y | U | Moderate |
| 16. | [20] | Y | Y | N | Y | Y | N | N | U | Y | Y | Moderate |
| 17. | [21] | Y | Y | N | Y | Y | U | Y | Y | Y | Y | High |
| 18. | [22] | Y | Y | Y | Y | Y | N | Y | Y | Y | Y | High |
| 19. | [23] | Y | Y | N | Y | Y | Y | Y | Y | Y | Y | High |
| 20. | [24] | Y | Y | N | Y | Y | N | Y | Y | Y | Y | High |
| 21. | [25] | Y | Y | Y | Y | Y | N | Y | Y | Y | Y | High |
| 22. | [26] | Y | Y | U | Y | Y | Y | Y | Y | Y | Y | High |
| 23. | [29] | Y | Y | N | Y | Y | N | Y | Y | Y | Y | High |
| 24. | [30] | Y | Y | N | Y | Y | N | Y | Y | Y | Y | High |
| 25. | [31] | Y | Y | N | Y | Y | N | Y | Y | Y | Y | High |
| 26. | [32] | Y | Y | N | Y | Y | N | Y | U | Y | Y | Moderate |
| 27. | [33] | Y | Y | N | Y | Y | N | Y | Y | Y | Y | High |
| 28. | [34] | Y | Y | N | Y | Y | N | Y | Y | Y | Y | High |
| 29. | [37] | Y | Y | N | Y | Y | N | Y | U | Y | Y | Moderate |
| 30. | [38] | Y | Y | N | Y | Y | N | Y | Y | Y | Y | High |
| 31. | [39] | Y | Y | N | N | Y | N | N | Y | Y | U | Moderate |
| 32. | [40] | Y | Y | N | Y | Y | N | Y | Y | Y | Y | High |
| 33. | [41] | Y | Y | N | Y | Y | N | Y | Y | Y | Y | High |
| 34. | [42] | Y | Y | N | Y | Y | N | Y | Y | Y | Y | High |
| 35. | [43] | Y | Y | Y | Y | Y | N | N | N | Y | Y | Moderate |
| 36. | [44] | Y | Y | N | Y | Y | N | Y | U | Y | Y | Moderate |
| 37. | [49] | Y | Y | N | Y | Y | N | Y | N | Y | Y | Moderate |
| 38. | [50] | Y | Y | Y | Y | Y | N | Y | Y | Y | Y | High |
| 39. | [52] | Y | Y | N | Y | Y | N | Y | Y | Y | Y | High |
| 40. | [53] | Y | Y | N | Y | Y | N | Y | Y | Y | Y | High |
| 41. | [55] | Y | Y | Y | Y | Y | N | Y | Y | Y | Y | High |
| 42. | [56] | Y | Y | N | Y | Y | N | Y | N | Y | Y | Moderate |
| 43. | [58] | Y | Y | N | Y | Y | N | Y | Y | Y | Y | High |
| 44. | [59] | Y | Y | N | Y | Y | Y | Y | U | Y | Y | High |
| 45. | [60] | Y | Y | N | Y | Y | Y | Y | Y | Y | Y | High |
| 46. | [61] | Y | Y | N | Y | Y | N | Y | Y | Y | Y | High |
| 47. | [63] | Y | Y | N | Y | Y | N | N | Y | Y | Y | Moderate |
| 48. | [65] | Y | Y | Y | Y | Y | U | Y | Y | Y | Y | High |
| 49. | [66] | Y | Y | N | Y | Y | N | Y | Y | Y | Y | High |
| 50. | [69] | Y | Y | N | Y | Y | N | Y | Y | Y | Y | High |
| 51. | [70] | Y | Y | Y | Y | Y | N | N | Y | Y | Y | High |
| Included mix-method publications | | | | | | | | | | | | |
| 1. | [2] | Y | Y | Y | Y | Y | N | Y | N | Y | Y | High |
| 2. | [4] | Y | Y | N | U | Y | U | Y | Y | Y | N | Moderate |
| 3. | [7] | Y | Y | N | U | Y | N | Y | N | Y | Y | Moderate |
| 4. | [17] | Y | Y | N | Y | Y | N | N | N | Y | Y | Moderate |
| 5. | [27] | Y | Y | U | Y | Y | N | N | Y | Y | Y | Moderate |
| 6. | [28] | Y | Y | N | Y | Y | N | Y | Y | Y | Y | High |
| 7. | [35] | Y | Y | N | Y | Y | N | U | N | Y | Y | Moderate |
| 8. | [36] | Y | Y | Y | Y | Y | N | Y | Y | Y | Y | High |
| 9. | [45] | Y | Y | N | Y | Y | N | Y | N | Y | U | Moderate |
| 10. | [46] | Y | Y | N | Y | Y | Y | Y | Y | Y | Y | High |
| 11. | [47] | Y | Y | N | Y | Y | N | Y | U | Y | Y | Moderate |
| 12. | [48] | Y | Y | N | Y | Y | N | Y | U | Y | Y | Moderate |
| 13. | [51] | Y | Y | N | Y | Y | N | Y | Y | Y | Y | High |
| 14. | [54] | Y | Y | N | Y | Y | N | Y | N | Y | Y | Moderate |
| 15. | [57] | Y | Y | N | Y | Y | N | Y | U | Y | Y | High |
| 16. | [62] | Y | Y | N | Y | U | N | Y | Y | Y | Y | Moderate |
| 17. | [64] | Y | Y | N | Y | Y | N | Y | Y | Y | Y | High |
| 18. | [67] | Y | Y | N | Y | Y | N | Y | N | Y | Y | Moderate |
| 19. | [68] | Y | Y | N | Y | Y | N | Y | Y | Y | Y | High |

^a^Critical Appraisal Skills Programme (2018). CASP (Qualitative) Checklist. [online] Available at: https://casp-uk.net/checklists/casp-qualitative-studies-checklist.pdf. Accessed: 06. 02.2024;

^b^ Y, yes; ^c^ N, no; ^d^ U, unclear.

**Reference List**

[1] Åkerlind C, Martin L, Gustafsson C. eHomecare and safety: The experiences of older patients and their relatives. Geriatric Nursing. 2018 Mar 1;39(2):178-85.

[2] Bajones M, Fischinger D, Weiss A, De La Puente P, Wolf D, Vincze M, Körtner T, Weninger M, Papoutsakis K, Michel D, Qammaz A, Panteleris P, Foukarakis M, Adami I, Ioannidi D, Leonidis A, Antona M, Argyros A, Mayer P, Panek P, Eftring H, Frennert S. Results of Field Trials with a Mobile Service Robot for Older Adults in 16 Private Households. Acm Transactions on Human-Robot Interaction. 2020;9(2):1-27.

[3] Baric V, Andreassen M, Öhman A, Hemmingsson H. Using an interactive digital calendar with mobile phone reminders by senior people - a focus group study. BMC Geriatrics. 2019; 19(116):1-11.

[4] Bevilacqua R, Felici E, Cavallo F, Amabili G, Maranesi E. Designing Acceptable Robots for Assisting Older Adults: A Pilot Study on the Willingness to Interact. Int J Environ Res Public Health. 2021;18(10686):1-9.

[5] Bian C, Bing Y, Hoonakker A, Mihailidis A. Attitudes and perspectives of older adults on technologies for assessing frailty in home settings: a focus group study. BMC Geriatrics. 2021;21(298): 1-13.

[6] Boström M, Kjellström S, Björklund A. Older persons have ambivalent feelings about the use of monitoring technologies. Technology & Disability. 2013;25(2): 117-125.

[7] Broadbent E, Tamagawa R, Patience A, Knock B. Attitudes towards health-care robots in a retirement village. Australasian Journal on Ageing. 2012;31(2): 115-120.

[8] Cabrita M, Tabak M, Vollenbroek-Hutten M. Older Adults' Attitudes Toward Ambulatory Technology to Support Monitoring and Coaching of Healthy Behaviors: Qualitative Study. JMIR Aging. 2019;2(1):1-12.

[9] Cajita MI, Hodgson NA, Lam KW, Yoo S, Han HR. Facilitators and Barriers to mHealth Adoption in Older Adults with Heart Failure. CIN: Computer, Informatics, Nursing. 2018;36(8):376-382.

[10] Chang CP, Lee TT, Mills ME.  Experience of Home Telehealth Technology in Older Patients With Diabetes. CIN: Computers, informatics, Nursing. 2017;35(10):530-537.

[11] Choi YK, Thompson HJ, Demiris G. Internet-of-Things Smart Home Technology to Support Aging-in-Place: Older Adults' Perceptions and Attitudes. Journal of Gerontological Nursing. 2021;47(4):15-21.

[12] Chung J, Thompson HJ, Joe J, Hall A, Demiris G. Examining Korean and Korean American older adults' perceived acceptability of home-based monitoring technologies in the context of culture. Informatics for Health & Social Care. 2017;42(1):61-76.

[13] Coghlan S, Waycott J, Lazar A, Barbosa Neves B. Dignity, Autonomy, and Style of Company: Dimensions Older Adults Consider for Robot Companions. Proc ACM Hum Comput Interact. 2021;5(CSCW1):1-24.

[14] Courtney KL. Privacy and senior willingness to adopt smart home information technology in residential care facilities. Methods of Information in Medicine. 2008;47(1):76-81.

[15] Courtney KL, Demeris G, Rantz M, Skubic M. Needing smart home technologies: the perspectives of older adults in continuing care retirement communities. Informatics in Primary Care. 2008;16:195-201.

[16] Daniele K, Marcucci M, Cattaneo C, Borghese NA, Zannini L. How Prefrail Older People Living Alone Perceive Information and Communications Technology and What They Would Ask a Robot for: Qualitative Study. Journal of Medical Internet Research. 2019;21(8):1-12.

[17] Davenport RD, Elzabadani H, Johnson JL, Helal AS, Mann WC. Pilot live-in trial at the GatorTech Smarthouse. Topics in Geriatric Rehabilitation. 2007;23(1):73-84.

[18] Demiris G, Rantz MJ, Aud MA, Marek KD, Tyrer HW, Skubic M, Hussam AA. Older adults' attitudes towards and perceptions of 'smart home' technologies: a pilot study,. Medical Informatics & the Internet in Medicine. 2004;29(2):87-94.

[19] Demiris G, Oliver DP, Dickey G, Skubic M, Rantz M. Findings from a participatory evaluation of a smart home application for older adults. Technol Health Care. 2008;16(2):111-118.

[20] Demiris G, Hensel BK, Skubic M, Rantz M. Senior residents' perceived need of and preferences for 'smart home' sensor technologies. International Journal of Technology Assessment in Health Care. 2008;24(1):120-124.

[21] Dermody G, Fritz R, Glass C, Dunham M, Whitehead L. Factors influencing community‐dwelling older adults’ readiness to adopt smart home technology: A qualitative exploratory study. Journal of Advanced Nursing. 2021 Dec;77(12):4847-61.

[22] Deutsch I, Erel H, Paz M, Hoffman G, Zuckerman O. Home robotic devices for older adults: Opportunities and concerns. Computers in Human Behavior. 2019;98:122-133.

[23] Ehn M, Eriksson LC, Åkerberg N, Johansson AC. Activity Monitors as Support for Older Persons' Physical Activity in Daily Life: Qualitative Study of the Users' Experiences. JMIR Mhealth and Uhealth. 2018;6(2):1-15.

[24] Ehn M, Johansson AC, Revenäs Å. Technology-Based Motivation Support for Seniors' Physical Activity-A Qualitative Study on Seniors' and Health Care Professionals' Views. International Journal of Environmental Research and Public Health. 2019;16(13):1-20.

[25] Elers P, Hunter I, Whiddett D, Lockhart C, Guesgen H, Singh A. User Requirements for Technology to Assist Aging in Place: Qualitative Study of Older People and Their Informal Support Networks. JMIR Mhealth and Uhealth. 2018;6(6):1-7.

[26] Essen A. The two facets of electronic care surveillance: an exploration of the views of older people who live with monitoring devices. Social Science&Medicine. 2008;67(1):128-136.

[27] Farivar S, Abouzahra M, Ghasemaghaei M. Wearable device adoption among older adults: A mixed-methods study. International Journal of Information Management. 2020;55:1-14.

[28] Fisher K, Easton K. The meaning and value of digital technology adoption for older adults with sight loss: A mixed methods study. Technology & Disability. 2019;30(4):177-184.

[29] Göransson C, Eriksson I, Ziegert K, Wengström Y, Langius‐Eklöf A, Brovall M, Kihlgren A, Blomberg K. Testing an app for reporting health concerns-Experiences from older people and home care nurses. International Journal of Older People Nursing. 2018;13(2):e12181.

[30] Harrefors C, Axelsson K, Sävenstedt S. Using assistive technology services at differing levels of care: healthy older couples' perceptions. Journal of Advanced Nursing. 2010;66(7):1523-1532.

[31] Harris MT, Rogers WA. Developing a Healthcare Technology Acceptance Model (H-TAM) for Older Adults with Hypertension. Ageing & Society. 2021;1-21.

[32] Hein Willius A, Torres Hidalgo M, Arroyo Zuñiga P, Quezada Venegas M, Arriagada Díaz C, Valenzuela Abarca E, San Martín Gutierrez E, Bedregal P. An Acceptability Study of A Personal Portable Device Storing Critical Health Information To Ensure Treatment Continuity Of Home-Dwelling Older Adults In Case Of A Disaster." Patient Prefer Adherence. 2019;13:1941-1949.

[33] Holender A, Sutton S, De Simoni A. Opinions on the use of technology to improve tablet taking in >65-year-old patients on cardiovascular medications. J Int Med Res. 2018;46(7):2754-2768.

[34] Holthe T, Halvorsrud L, Lund A. A critical occupational perspective on user engagement of older adults in an assisted living facility in technology research over three years. Journal of Occupational Science. 2020;27(3):376-389.

[35] Huang TY, Huang C. Elderly's acceptance of companion robots from the perspective of user factors. Universal Access in the Information Society. 2020;19:935-948.

[36] Hvalic-Touzery S, Smole-Orehek K, Dolnicar V. Exploring reciprocity in perceptions on telecare within the informal carer-care receiver dyad. Teorija in Praksa. 2021;58(3):840-859.

[37] Jo TH, Ma JH, Cha SH. Elderly Perception on the Internet of Things-Based Integrated Smart-Home System. Sensors. 2021;21(1284):1-28.

[38] Johnson A, Shukla N, Halley M, Nava V, Budaraju J, Zhang L, Linos E. Barriers and facilitators to mobile health and active surveillance use among older adults with skin disease. Health Expectations. 2021;24:1582-1592.

[39] Kärki A, Sallinen M, Kuusinen J. How to live independently with or without technology? Stud Health Technol Inform. 2015;217:306-310.

[40] Knowles B, Hanson VL. Older Adults' Deployment of 'Distrust'. ACM Transactions on Computer-Human Interaction. 2018;25(4):1-25.

[41] Kononova A, Li L, Kamp K, Bowen M, Rikard RV, Cotton S, Peng W. The Use of Wearable Activity Trackers Among Older Adults: Focus Group Study of Tracker Perceptions, Motivators, and Barriers in the Maintenance Stage of Behavior Change. JMIR Mhealth Uhealth. 2019;7(4):1-16.

[42] Ladin K, Porteny T, Perugini JM, Gonzales KM, Aufort KE, Levine SK, Wong JB, Isakova T, Rifkin D, Gordon EJ, Rossi A, Koch-Weser S, Weiner DE. Perceptions of Telehealth vs In-Person Visits Among Older Adults With Advanced Kidney Disease, Care Partners, and Clinicians. JAMA Network Open. 2021;4(12):1-12.

[43] Leikas J, Kulju M. Ethical consideration of home monitoring technology: A qualitative focus group study. Gerontechnology. 2018;17(1):38-47.

[44] Lie MLS, Lindsay S, Brittain K. Technology and trust: older people's perspectives of a home monitoring system. Ageing & Society. 2016;75(3):1-25.

[45] McGlynn SA, Kemple SC, Mitzner TL, King CH, Rogers WA. Understanding Older Adults' Perceptions of Usefulness for the Paro Robot. Proc Hum Factors Ergon Soc Annu Meet. 2014;58(1):1914-1918.

[46] McGlynn SA, Kemple S, Mitzner TL, King CHA, Rogers WA. Understanding the potential of PARO for healthy older adults. International Journal of Human-Computer Studies. 2017;100:33-47.

[47] Neves BB, Mead G. Digital Technology and Older People: Towards a Sociological Approach to Technology Adoption in Later Life. Sociology-the Journal of the British Sociological Association. 2021;55(5):888-905.

[48] Neves BB, Waycott J, Maddox A. When Technologies are Not Enough: The Challenges of Digital Interventions to Address Loneliness in Later Life. Sociological Research Online. 2023;28(1):150-170.

[49] Niemela M, van Aerschot L, Tammela A, Aaltonen, Lammi H. Towards Ethical Guidelines of Using Telepresence Robots in Residential Care. International Journal of Social Robotics. 2021;13(3):431-439.

[50] Ostrowski AK, Harrington CN, Breazeal C, Won Park H. Personal Narratives in Technology Design: The Value of Sharing Older Adults' Stories in the Design of Social Robots. Frontiers in Robotics and AI. 2021;8:1-17.

[51] Park YH, Chang HK, Lee MH, Lee SH. Community-dwelling older adults' needs and acceptance regarding the use of robot technology to assist with daily living performance. BMC Geriatrics. 2019;19(208):1-9.

[52] Peek STM, Luijkx KG, Rijnaard MD, Nieboer ME, van der Voort CS, Aarts S, van Hoof J, Vrijhoef HJM, Wouters EJM. Older Adults' Reasons for Using Technology while Aging in Place. Gerontology. 2016;62(2):226-237.

[53] Peek STM, Luijkx KG, Vrijhoef HJM, Nieboer ME, Aarts S, van der Voort CS, Rijnaard MD, Wouters EJM. Understanding changes and stability in the long-term use of technologies by seniors who are aging in place: a dynamical framework. BMC Geriatrics. 2019;19(236):1-13.

[54] Pigini L, Facal D, Blasi L, Andrich R. Service robots in elderly care at home: Users' needs and perceptions as a basis for concept development. Technology & Disability. 2012;24(4):303-311.

[55] Pol M, van Nes F, van Hartingsveldt M, Buurman B, de Rooij S, Kröse B. Older People's Perspectives Regarding the Use of Sensor Monitoring in Their Home. The Gerontologist. 2016;56(3):485-493.

[56] Portet F, Vacher M, Golanski C, Roux C, Meillon B. Design and evaluation of a smart home voice interface for the elderly: acceptability and objection aspects. Personal and Ubiquitous Computing. 2013;17:127-144.

[57] Pripfl J, Körtner T, Batko-Klein D, Hebesberger D, Weninger M, Gisinger C. Social service robots to support independent living Experiences from a field trial. Zeitschrift Fur Gerontologie Und Geriatrie. 2016;49:282-287.

[58] Sanchez VG, Anker-Hansen C, Taylor I, Eilertsen G. Older People's Attitudes and Perspectives Of Welfare Technology In Norway. Journal of Multidisciplinary Healthcare. 2019;12:841-853.

[59] Stack E, King R, Janko B, Burnett M, Hammersley N, Agarwal V, Hannuna S, Burrows A, Ashburn A. Could In‐Home Sensors Surpass Human Observation of People with Parkinson’s at High Risk of Falling? An Ethnographic Study. BioMed research international. 2016;2016:1-10.

[60] Steele R, Lo A, Secombe C, Wong YK. Elderly persons’ perception and acceptance of using wireless sensor networks to assist healthcare. International journal of medical informatics. 2009 Dec 1;78(12):788-801.

[61] Thilo FJ, Bilger S, Halfens RJG, Schols JMGA, Hahn S. Involvement of the end user: exploration of older people's needs and preferences for a wearable fall detection device - a qualitative descriptive study. Patient Prefer Adherence. 2017;11:11-22.

[62] Thomas L, Little L, Briggs P, McInnes L, Jones E, Nicholson J. Location tracking: views from the older adult population. Age and ageing. 2013 Nov 1;42(6):758-63.

[63] Tsai HS, Shillair R, Cotton SR, Winstead V, Yost E. Getting Grandma Online: Are Tablets the Answer for Increasing Digital Inclusion for Older Adults in the US? Educational Gerontology. 2015;41(10): 695-709.

[64] Van Houwelingen CTM, Ettema RGA, Antonietti MGEF, Kort HSM. Understanding Older People's Readiness for Receiving Telehealth: Mixed-Method Study. Journal of Medical Internet Research. 2018;20(4): 1-17.

[65] Vandemeulebroucke T, Dierckx de Casterlé B, Welbergen L, Massart M, Gastmans C. The ethics of socially assistive robots in aged care. A focus group study with older adults in Flanders, Belgium. The Journals of Gerontology: Series B. 2020 Nov;75(9):1996-2007.

[66] Vaportzis E, Clausen MG, Gow AJ. Older Adults Perceptions of Technology and Barriers to Interacting with Tablet Computers: A Focus Group Study. Frontiers in Psychology. 2017;8(1687):1-11.

[67] Walsh K, Callan A. Perceptions, Preferences, and Acceptance of Information and Communication Technologies in Older-Adult Community Care Settings in Ireland: A Case-Study and Ranked-Care Program Analysis. Ageing International. 2011;36: 102-122.

[68] Wang SZ, Bolling K, Mao W, Reichstadt J, Jeste D, Kim HC, Nebeker C. Technology to Support Aging in Place: Older Adults' Perspectives. Healthcare. 2019;7(60):1-18.

[69] Wild K, Boise L, Lundell J, Foucek A. Unobtrusive in-home monitoring of cognitive and physical health: reactions and perceptions of older adults. Journal of Applied Gerontology. 2008;27(2):181-200.

[70] Wilson-Nash C, Tinson J. 'I am the master of my fate': digital technology paradoxes and the coping strategies of older consumers. Journal of Marketing Management. 2022;38(3-4):248-278.
